# Supplementary material for: A genome‐scale screen reveals context‐dependent ovarian cancer sensitivity to miRNA overexpression
Source: Mol Syst Biol. 2015 Dec 11;11(12):842. doi: 10.15252/msb.20156308 (PMC4704493; doi:10.15252/msb.20156308)
Supplement: Supplementary file 1 — Expanded View Figures PDF [file MSB-11-842-s001.pdf]

Expanded View Figures

Figure EV1. Primary screen data analytics.

A Transfection conditions were optimized using normalized cell viability values upon transfection of mimic negative control and a pan-toxic siRNA, siUBB. Values plotted as the mean of three separate experiments with the bars representing the range.

B For each microRNA mimic, a standard deviation value was calculated for mean viability across the panel of 16 cell lines (blue curve). In addition, a within-miRNA seed family standard deviation value was calculated for mean viability across the cell panel (red curve). Lastly, a within-replicate standard deviation value was calculated (black curve). A kernel density estimation was fit to each of the three standard deviation distributions and plotted.

C The indicated microRNA mimics were clustered, using hierarchical APC, based on their viability *z*-scores across 16 cell lines. A Euclidean distance metric was used. Node colors indicate cluster membership. A high-resolution searchable and zoomable pdf has been provided as Dataset EV11 to facilitate visualization of cluster entities.

D The indicated ovarian cancer cell lines were clustered, using hierarchical APC, according to miR mimic viability *z*-scores (400 miRs). Node colors indicate cluster membership.

E RNASeq data for the indicated cell lines (acquired from the CCLE or internal analysis (this study)) was first filtered to select the top 20% of the most highly variant genes (2,686 genes total). The cell lines were then clustered using hierarchical APC based on a Euclidean distance metric. Node colors indicate cluster membership.

F Nodes in the gene expression-based APC from (E) were relabeled according to their cluster membership in the miR mimic response-based clusters from (D).

© 2015 The Authors

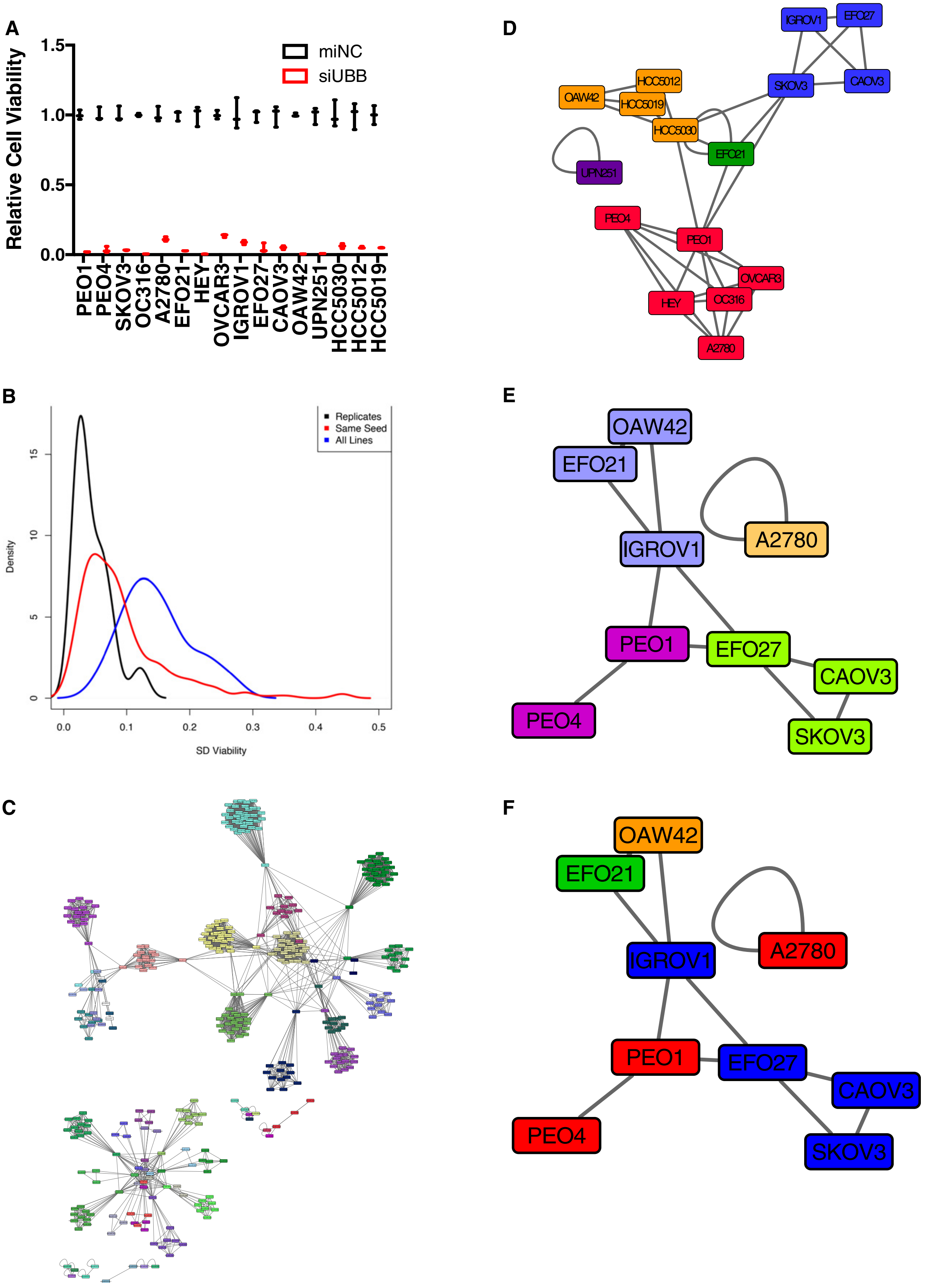

Figure EV1.

 © 2015 The Authors

Figure EV2. Increased expression of miR-146a and miR-505 correlates with increased patient survival.

A A Tukey plot of the range of *z*-scores for all mimics screened in each cell line screened. *Z*-scores are representative of the mean viability of 3 replicates.

B Quantitative PCR (qPCR) analysis of endogenous miR-146a expression revealed no association between level of expression in a cell line and toxicity in the screen (top panel). To facilitate line-to-line comparison, endogenous expression values were normalized to a reference cell line (Hey cells). qPCR showed similar levels of overexpression among all cell lines (bottom panel). Bars in red display the endogenous expression of miR-146a (normalized to RNU6B using the comparative CT method), while bars in black display the overexpression of miR-146a mimic after transfection (normalized similar to endogenous expression).

C qPCR analysis of endogenous miR-505 expression revealed no association between level of expression in a cell line and toxicity in the screen (top panel). To facilitate line-to-line comparison, endogenous expression values were normalized to a reference cell line (Hey cells). qPCR showed similar levels of overexpression among all cell lines (bottom panel). Endogenous and overexpression values displayed as in (B). Bars represent the mean of 2 replicates $\pm$ SD.

D Kaplan–Meier plots of training and validation sets of tumors using miRNA expression from both Agilent arrays and Illumina miRseq showed that higher expression of miR-505 correlated with increased patient survival.

E Kaplan–Meier plots of training and validation sets of tumors using miRNA expression from both Agilent arrays and Illumina miRseq showed that higher expression of miR-146a correlated with increased patient survival.

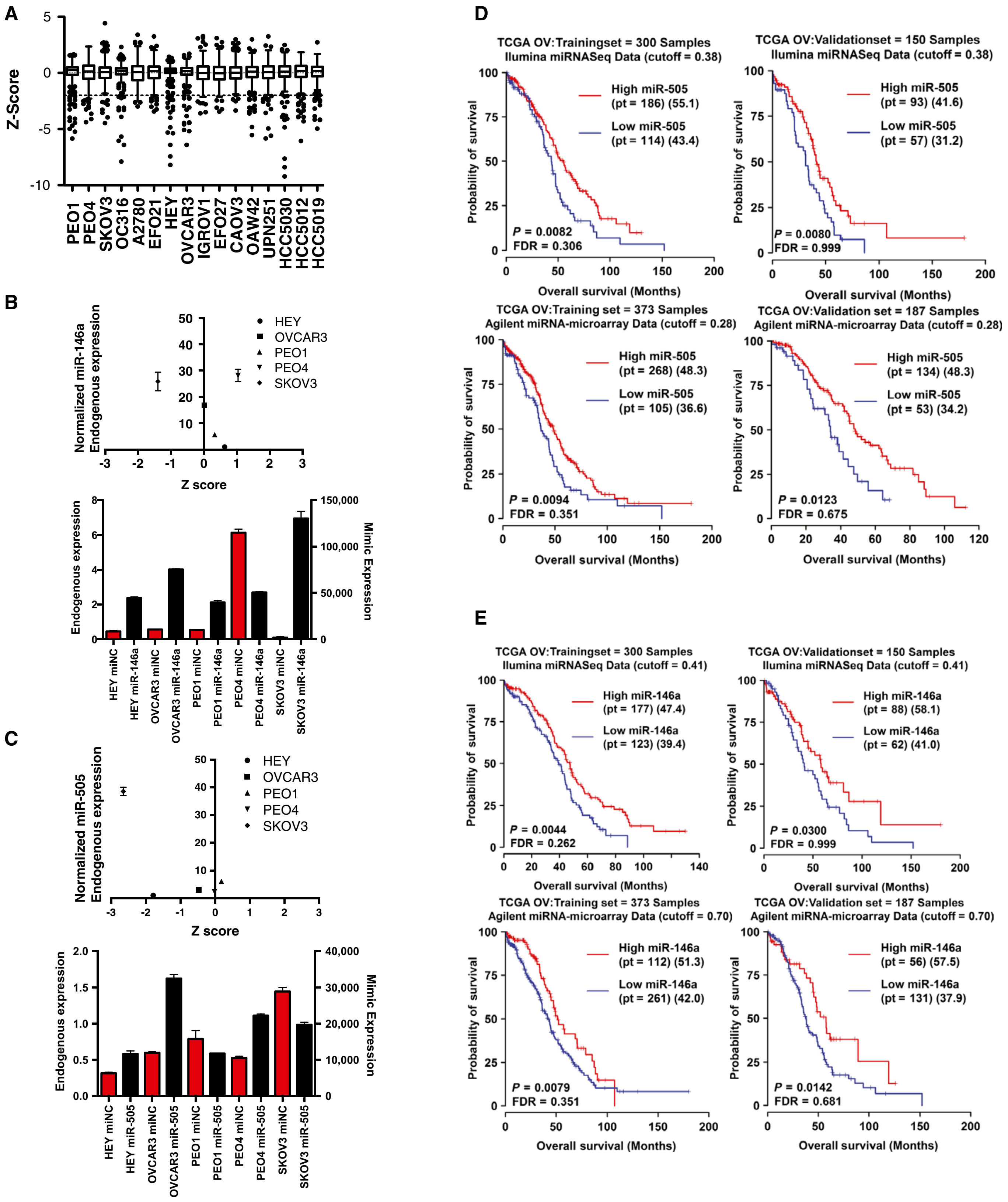

Figure EV2.

© 2015 The Authors

Figure EV3. Distinct miRNA sensitivities in patient-matched cell lines from recurrent disease.

A A schematic depicting the derivation of the PEO1 and PEO4 cell lines from a patient with high-grade serous adenocarcinoma of the ovary.

B A scatter plot of *z*-scores corresponding to inhibition of viability in PEO1 versus PEO4 in response to each of 400 miRNA mimics. Values were derived from the mean of triplicate experiments. Mimics with a *z*-score < 2 were considered to have significantly reduced viability in a cell line. 17 mimics reduced PEO1 cell viability, 15 mimics reduced PEO4 viability, and 2 mimics reduced viability in both cell lines.

C The scatter plot indicates the expression value of each miRNA in PEO1 (*y*-axis) and the effect of each miRNA on cell viability in the same cell line as represented by *z*-score (*x*-axis).

D The scatter plot indicates the expression value of each miRNA in PEO4 (*y*-axis) and the effect of each miRNA on cell viability in the same cell line as represented by *z*-score (*x*-axis).

E The scatter plots indicate the expression values of each miRNA in PEO1 (*x*-axis) and PEO4 (*y*-axis) (left panel), and miRNA expression relative to that in HOSE (Normal) cells (right panel). Points are color-coded according to the corresponding miRNA mimic activity in the viability screens.

F PEO1-specific hit miR-210 continues to show a robust phenotype at even a 10-fold dilution while increasing the dosage 4-fold does not sensitize PEO4 cells, suggesting that selectivity was not due to overt dosage effects. Each data point represents the mean of 3 independent experiments ± SD.

Source data are available online for this figure.

© 2015 The Authors

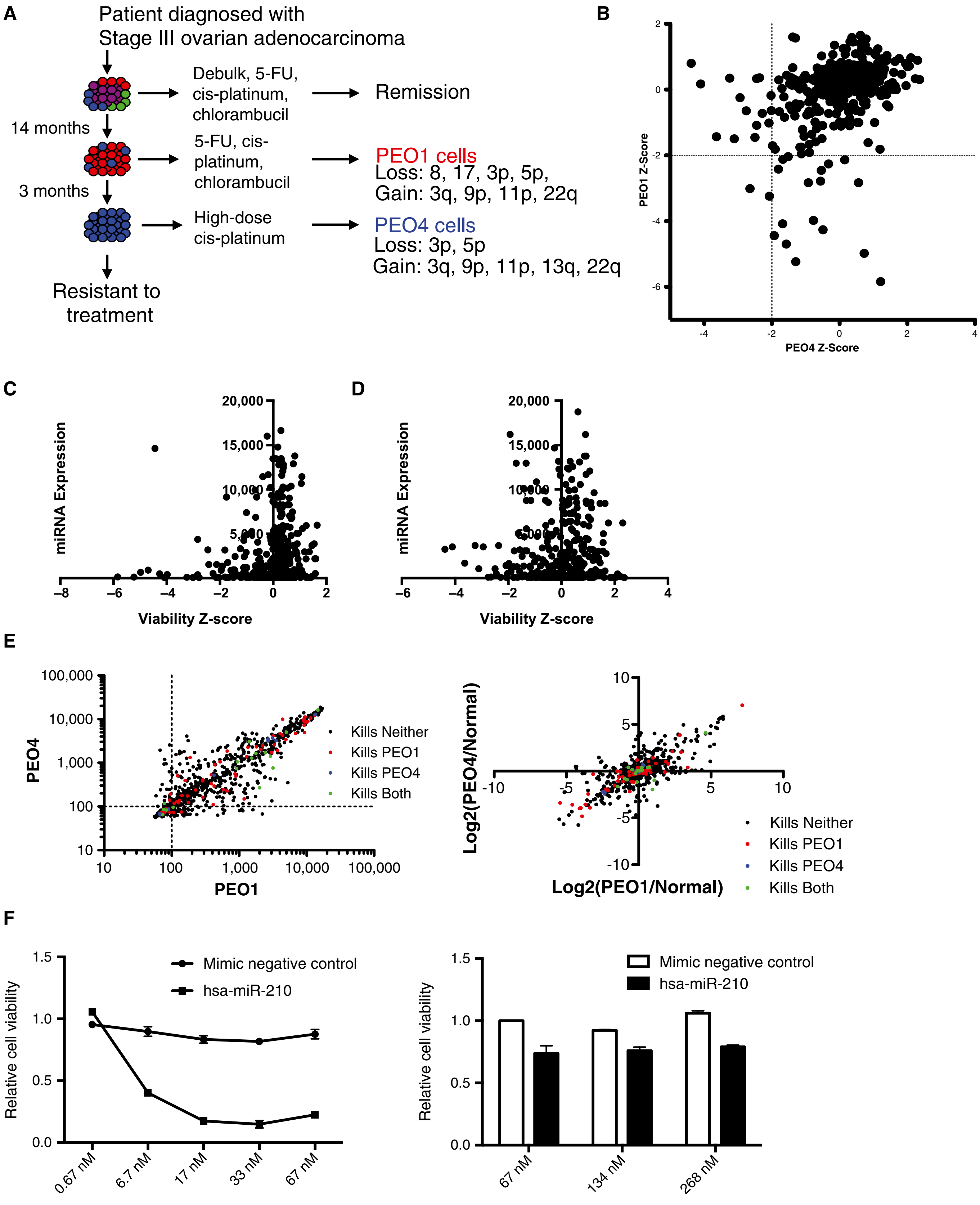

Figure EV3.

 © 2015 The Authors

Figure EV4. Genomic characterization of patient-matched cell lines from recurrent disease.

A A summary of the PEO1 and PEO4 genomic characterization is displayed by circos plot. The outer track indicates relative expression of genes (the $\log_2$ of the ratio of (RPKM+1) values of PEO1 versus PEO4). Red peaks correspond to genes with greater than two-fold overexpression in PEO1 cells, and blue peaks correspond to genes with greater than two-fold overexpression in PEO4 cells. The next three tracks represent SNVs unique to PEO1 in red, unique to PEO4 in blue, and common in black. This is followed by an axis indicating chromosome position. The inner four tracks summarize CNV as indicated (variation is defined by a read depth ratio of ≥ 1.5 (gain) or ≤ 0.5 (loss) as compared to the reference cell line). See Datasets EV2, EV3, and EV4 for detailed annotations.

B Principal component analysis (PCA) plot of 3 PEO1 (blue squares) and 3 PEO4 (pink squares) replicates based on data from genes with RPKM value of one or greater. EV, Eigen value.

© 2015 The Authors

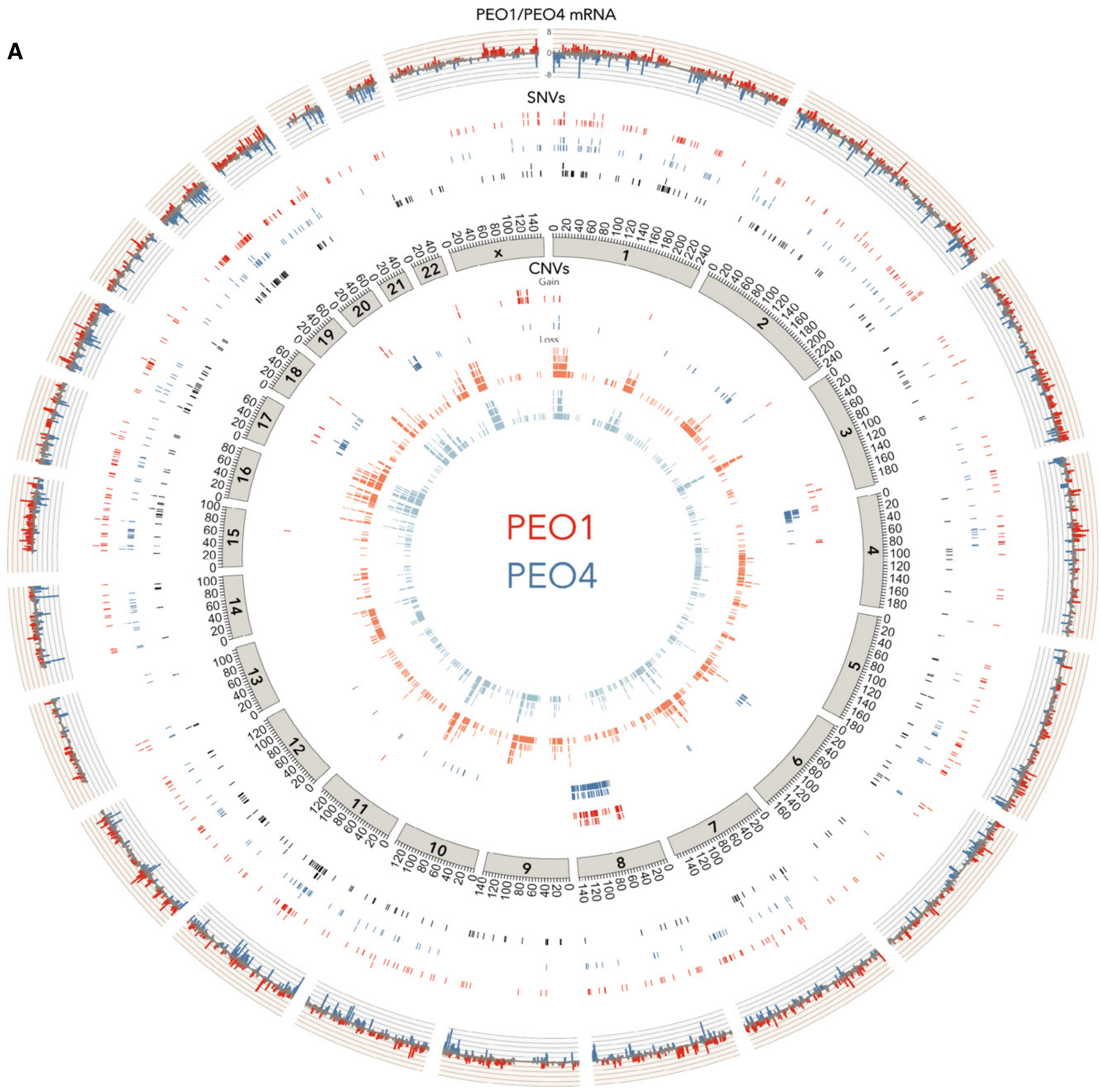

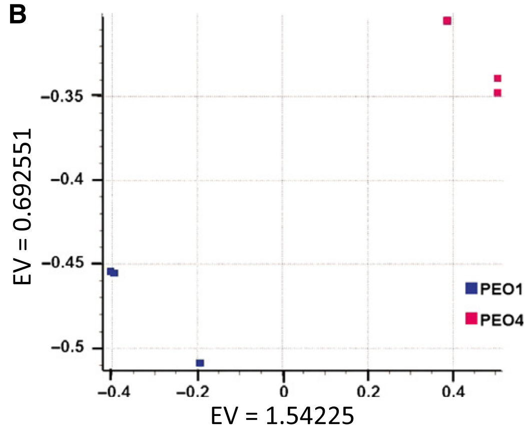

Figure EV4.

 © 2015 The Authors

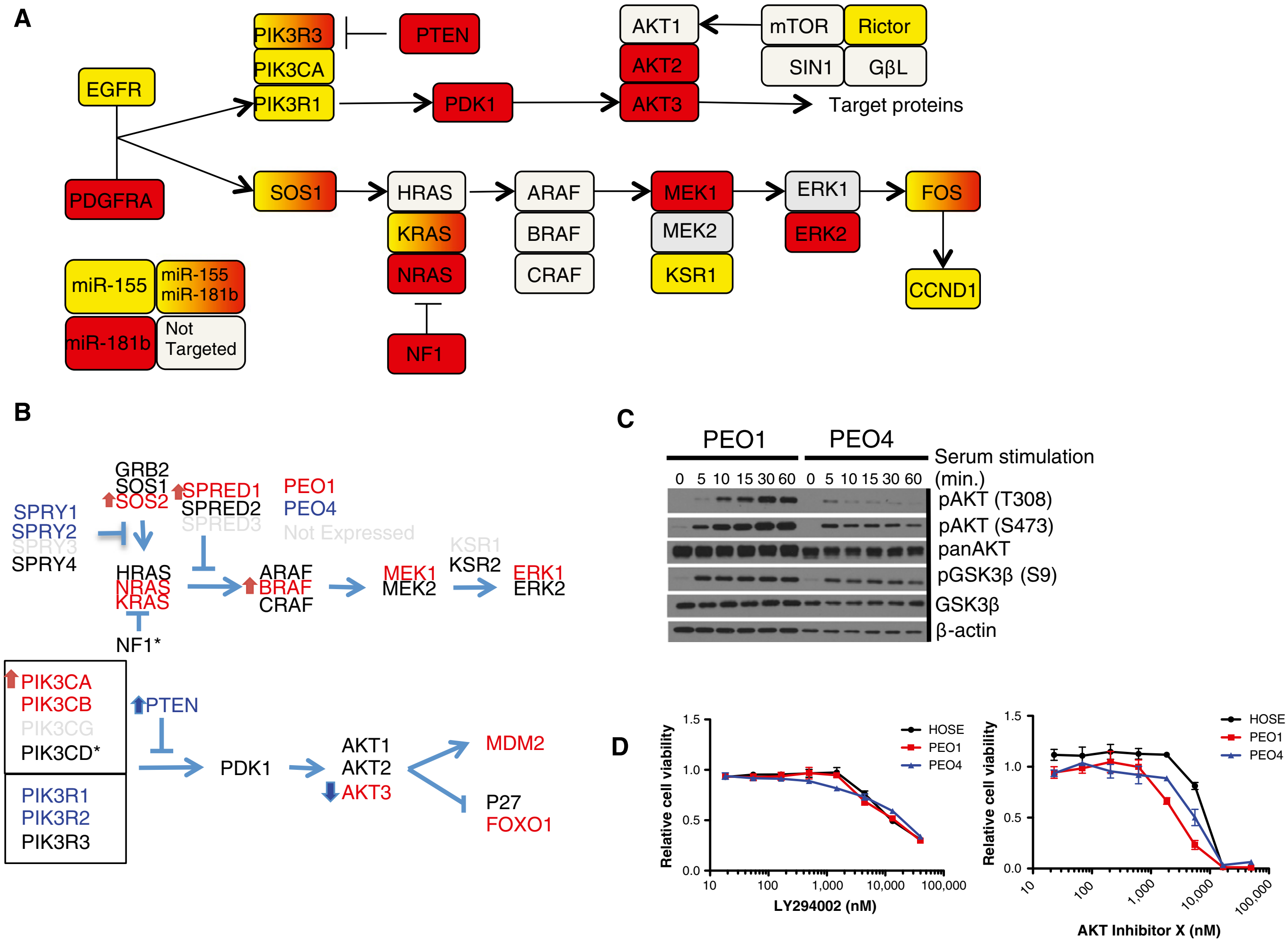

Figure EV5. miR-155 and miR-181b are predicted to target the AKT, MAPK, and TGFβ pathways.

A miR-155 and miR-181b are predicted to target multiple nodes in the AKT and MAPK signaling pathways. Nodes predicted to be targeted by miR-155 are in yellow, miR-181b in red, those predicted to be targeted by both are in yellow-to-red gradient, and those predicted to be targeted by neither are in gray. Note only miR-155 is predicted to target Rictor, which is part of the complex responsible for phosphorylation of AKT at S473.

B Genomic alterations predicted to influence AKT and MAPK signaling pathways in PEO1 and PEO4 cells. Genes in red were overexpressed at least 2-fold in PEO1 relative to PEO4 cells, while genes in blue were overexpressed in PEO4 relative to PEO1. Arrows indicated copy gain or loss with red indicating PEO1 cells and blue indicating PEO4. Asterisks denote genes containing SNVs.

C Immunoblots from whole-cell lysates indicate decreased AKT phosphorylation at both T308 and S473 in PEO4 cells relative to PEO1 cells in response to serum stimulation of cells deprived of serum overnight.

D Dose-dependent consequences of exposure to the PI3K inhibitor LY294002 or AKT Inhibitor X on cell viability was examined in the indicated cell lines. All values were normalized to DMSO carrier alone. Each point represents the mean of 3 experiments ± SD. No significant differences were detected.

Source data are available online for this figure.

© 2015 The Authors

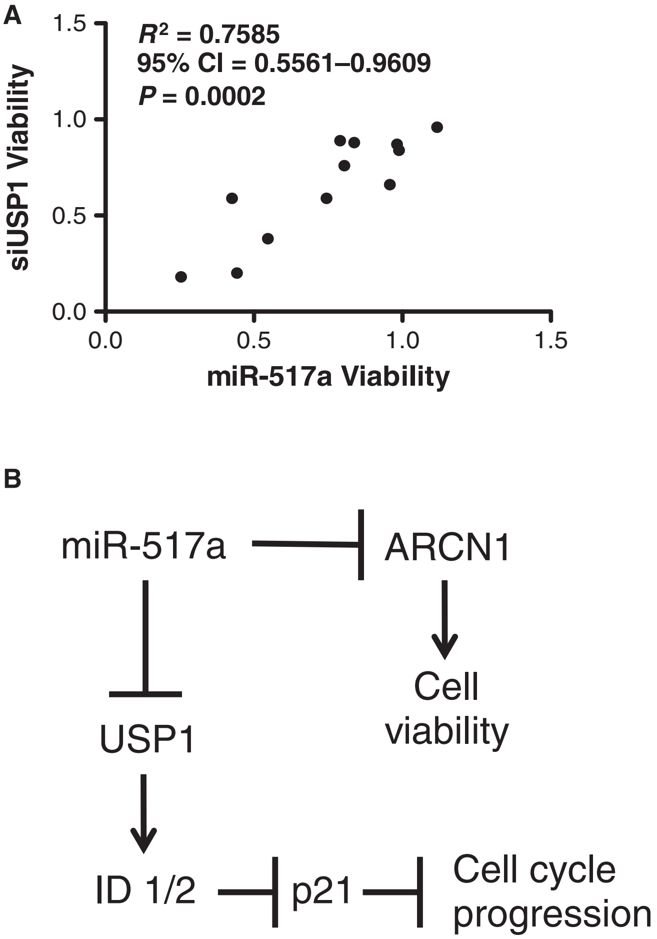

Figure EV6. miR-517a reduces ovarian cancer cell viability, in part, by inhibiting ARCN1 expression.

A Correlation of the consequence of USP1 depletion and miR-517a sensitivity in 12 NSCLC cell lines (plot as in Fig 2F).

B Schematic summarizing predicted miR-517a relationships with ARCN1 and USP1.

Source data are available online for this figure.

Figure EV7. Identification of miR-124-responsive genes.

A Expression of miR-124 mimic reduced expression of PTBP1 (PTB) and CTDSP1 (SCP1) but not other members of the REST complex on a microarray. Each bar represents the log_2 of the ratio of the means of three experiments of mir-124 mimic expression to mimic negative control expression of a single probe on the microarray.

B miR-124 mimic expression reduced expression of mesenchymal markers ITGB1, VIM, and TWIST2 and increased expression of neuronal markers CDH2, VAMP2, and MAP2 according to the microarray data. Each bar represents the log_2 of the ratio of the means of three experiments of mir-124 mimic expression to mimic negative control expression of a single probe on the microarray.

C SIX4, EYA1, EYA2, EYA3, and EYA4 are predicted targets of miR-124 in TargetScan (www.targetscan.org).

D SIX4 expression is significantly enriched in human ovarian tumors relative to normal ovarian tissue. *P*-value from Welch two-sample *t*-test. N, unmatched normal ovarian tissue; P, primary epithelial ovarian tumors; R, recurrent epithelial ovarian tumors.

E Knockdown of SIX4 reduced cell viability similar to expression of a miR-124 mimic in PEO1 cells (mean ± SD of 3 experiments).

F Expression of a SIX4 construct that cannot be targeted by miR-124 partially rescues toxicity due to miR-124 over expression in PEO1 cells. Mean ± SD of 6 experiments. Two-way ANOVA analysis, ***$P < 0.005$.

G SIX4 knockdown did not induce activation of caspase-3 and caspase-7. Depletion of SIX4 reduced BrdU incorporation in ES2 cells and resulted in decreased Hoechst-positive nuclei 48 h post-transfection. SIX4 knockdown also resulted in decreased cells in S-phase as seen in PI-stained ES2 cells 48 h post-transfection. All bars represent the mean ± SD of three experiments.

H Knockdown of SIX4 reduced expression of multiple cyclin proteins in PEO1 and PEO4 cells on the microarray. Each bar represents the log_2 of the ratio of the means of three experiments of SIX4 siRNA transfection to siRNA negative control transfection of a single probe on the microarray.

I SIX4 knockdown induced expression of STRADB in PEO1 and PEO4 cells according to the microarray data. Each bar represents the log_2 of the ratio of the means of three experiments of SIX4 siRNA transfection to siRNA negative control transfection of a single probe on the microarray.

J Schematic of predicted interactions of miR-124 with cell cycle and cell differentiation machinery.

Source data are available online for this figure

▶

 © 2015 The Authors

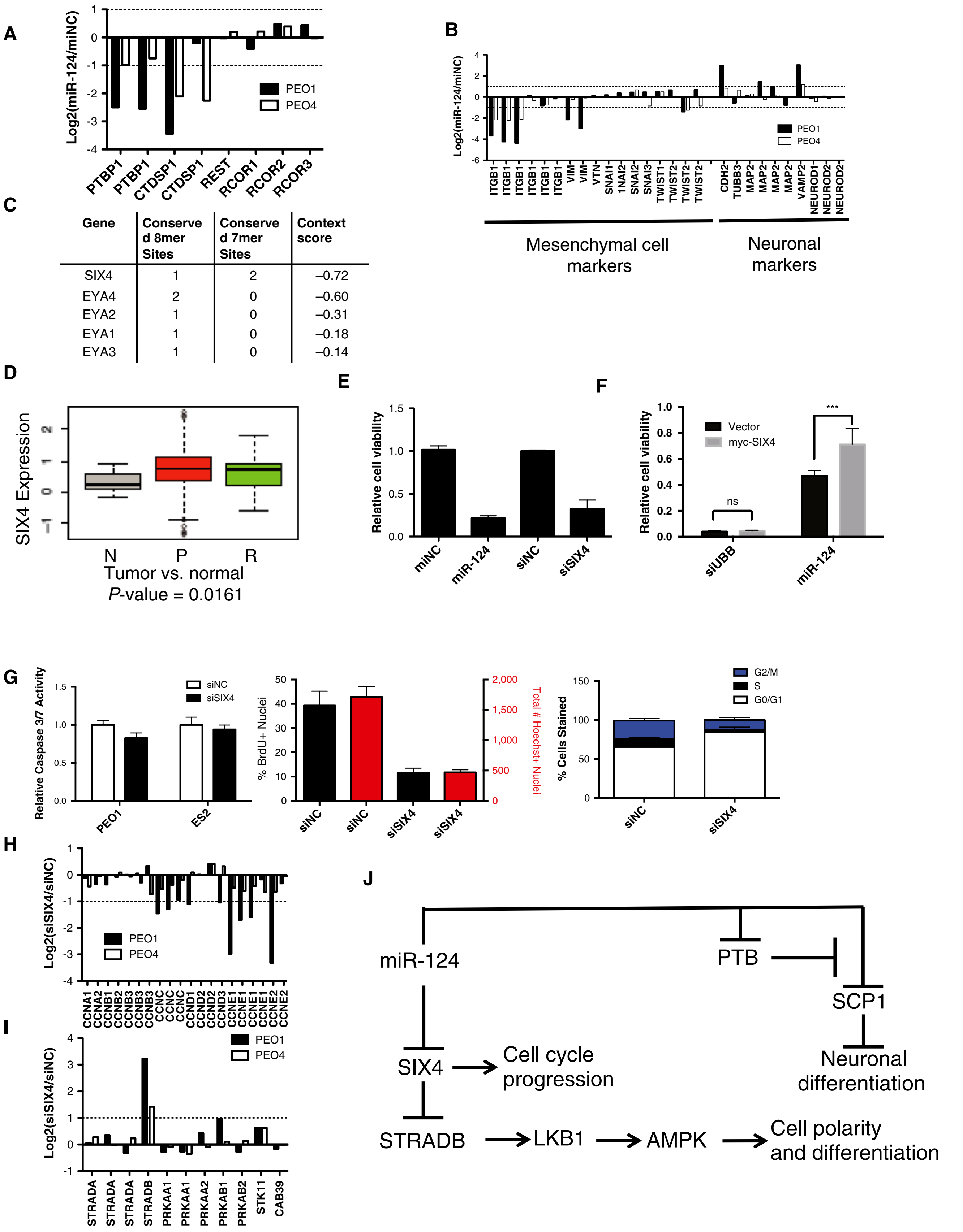

Gene	Conserved 8mer Sites	Conserved 7mer Sites	Context score
SIX4	1	2	–0.72
EYA4	2	0	–0.60
EYA2	1	0	–0.31
EYA1	1	0	–0.18
EYA3	1	0	–0.14

Figure EV7.

© 2015 The Authors
