# Supplementary material for: A genome‐scale screen reveals context‐dependent ovarian cancer sensitivity to miRNA overexpression
Source: Mol Syst Biol. 2015 Dec 11;11(12):842. doi: 10.15252/msb.20156308 (PMC4704493; doi:10.15252/msb.20156308)
Supplement: Supplementary file 2 — Table EV1 [file MSB-11-842-s002.docx]

**Table EV1. Cell Lines Screened and Optimal Transfection Conditions**

| **Cell Line** | **Comment** | **Transfection Reagent** | **Cells/Well** |
| --- | --- | --- | --- |
| A2780 | Established line | RNAiMax | 20,000 |
| CAOV3 | Established line | RNAiMax | 5000 |
| EFO21 | Established line | RNAiMax | 5000 |
| EFO27 | Established line | RNAiMax | 15,000 |
| HCC5012 | Low-passage non-clonal line | RNAiMax | 3000 |
| HCC5019 | Low-passage non-clonal line | RNAiMax | 5000 |
| HCC5030 | Low-passage non-clonal line | RNAiMax | 5000 |
| HEY | Established line | RNAiMax | 5000 |
| IGROV1 | Established line | RNAiMax | 12,500 |
| OAW42 | Established line | RNAiMax | 10,000 |
| OC316 | Established line | RNAiMax | 5000 |
| OVCAR3 | Established line | RNAiMax | 12,500 |
| PEO1 | Matched pair chemo-response/resistant | RNAiMax | 10,000 |
| PEO4 | Matched pair chemo-response/resistant | Dharmafect 3 | 10,000 |
| SKOV3 | Established line | RNAiMax | 5,000 |
| UPN251 | Established line | RNAiMax | 10,000 |
