# Supplementary material for: A genome‐scale screen reveals context‐dependent ovarian cancer sensitivity to miRNA overexpression
Source: Mol Syst Biol. 2015 Dec 11;11(12):842. doi: 10.15252/msb.20156308 (PMC4704493; doi:10.15252/msb.20156308)
Supplement: Supplementary file 3 — Table EV2 [file MSB-11-842-s003.docx]

**Table EV2. miRNA mimic and siRNA oligo sequences**

| Sequences: |
| --- |
| hsa-miR-210 – |
| 5’-CUGUGCGUGUGACAGCGGCUGA-3’ |
| hsa-miR-155 – |
| 3’-UUAAUGCUAAUCGUGAUAGGGGU-3’ |
| hsa-miR-181b – |
| 3’-AACAUUCAUUGCUGUCGGUGGGU-3’ |
| hsa-miR-517a – |
| 3’-AUCGUGCAUCCCUUUAGAGUGU-3’ |
| hsa-miR-124 – |
| 3’-UAAGGCACGCGGUGAAUGCC-3’ |
| PTEN – |
| 5’-GUGAAGAUCUUGACCAAUG-3’ |
| 5’-GAUCAGCAUACACAAAUUA-3’ |
| 5’-GGCGCUAUGUGUAUUAUUA-3’ |
| 5’-GUAUAGAGCGUGCAGAUAA-3’ |
| AKT3 – |
| 5’-GAAAGAUUGUGUACCGUGA-3’ |
| 5’-GGACUACUGUUAUAGAGAG-3’ |
| 5’-UGAGACAGAUACUAGAUAU-3’ |
| 5’-GCUCAUUCAUAGGAUAUAA-3’ |
| SMURF2 – |
| 5’-GAUGAGAACACUCCAAUUA-3’ |
| 5’-GACCAUACCUUCUGUGUUG-3’ |
| 5’-CAAAGUGGAAUCAGCAUUA-3’ |
| 5’-GAACAACACAAUUUACAGA-3’ |
| SMAD2 – |
| 5’-GAACAAACCAGGUCUCUUG-3’ |
| 5’-GCAGAACUAUCUCCUACUA-3’ |
| 5’-GAAGAGGAGUGCGCUUAUA-3’ |
| 5’-GGUGUUCGAUAGCAUAUUA-3’ |
| SMAD3 – |
| 5’-UCAAGAGCCUGGUCAAGAA-3’ |
| 5’-GAGUUCGCCUUCAAUAUGA-3’ |
| 5’-GGACGCAGGUUCUCCAAAC-3’ |
| 5’-GGACGAGGUCUGCGUGAAU-3’ |
| USP1 – |
| 5’-GCAUAGAGAUGGACAGUAU-3’ |
| 5’-GAAAUACACAGCCAAGUAA-3’ |
| 5’-CAUAGUGGCAUUACAAUUA-3’ |
| 5’-GCACAAAGCCAACUAACGA-3’ |
| SIX4 – |
| 5’-CCAGUGGAGUUAUCCUUAA-3’ |
| 5’-GAUGGAGGGUCUGUAGUGA-3’ |
| 5’-UGUCUUAGAUGGCAUGGUU-3’ |
| 5’-GUAUACACGGUUCCUAAUA-3’ |
| Animal Experiments |
| SIX4 from Sigma – |
| SASI_Mm01_00078274 5’-CUUAAUGAUGCUGGACUCU-3’ |
