# Supplementary figures and images for: A genome‐scale screen reveals context‐dependent ovarian cancer sensitivity to miRNA overexpression
Source: Mol Syst Biol. 2015 Dec 11;11(12):842. doi: 10.15252/msb.20156308 (PMC4704493; doi:10.15252/msb.20156308)

# Uncut Blots for figure EV5C

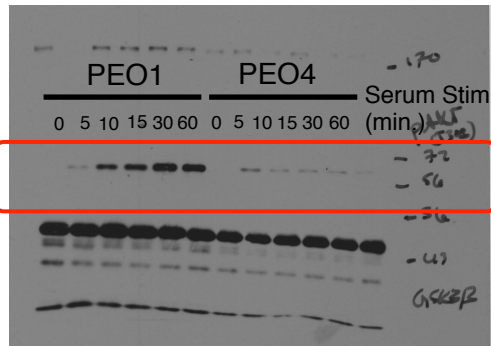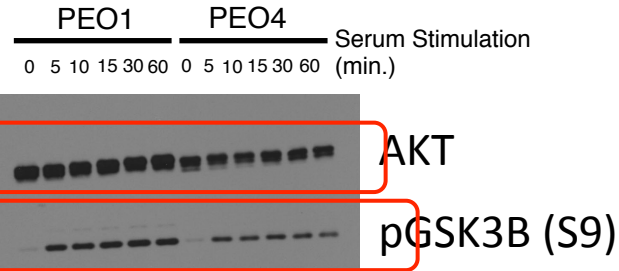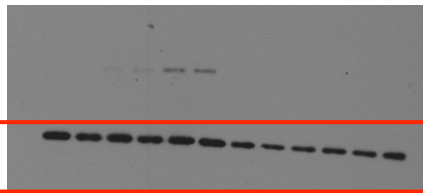

pAKT (T308)

GSK3B

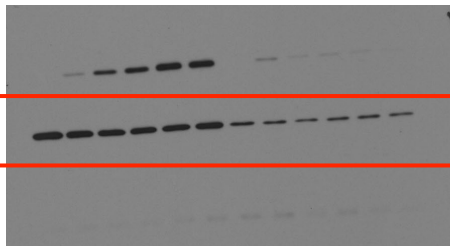

pAKT (S473)

beta-actin

p27

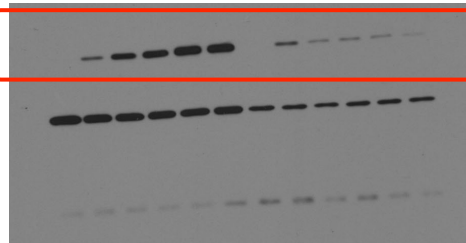

pAKT (S473)

beta-actin

p27

Supplement: Supplementary file 4 — Source Data for Expanded View [file MSB-11-842-s004.zip › Source_Data_EV/Source Data Figure EV5C.pdf]

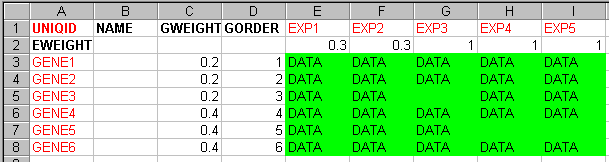

Supplement: Supplementary file 12 — Dataset EV8 [file MSB-11-842-s016.zip › Dataset_EV8/Cluster.app/Contents/Resources/format.bmp]

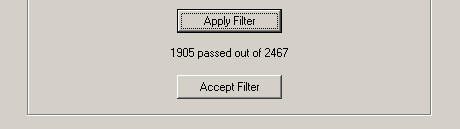

Supplement: Supplementary file 12 — Dataset EV8 [file MSB-11-842-s016.zip › Dataset_EV8/Cluster.app/Contents/Resources/html/images/accept.png]

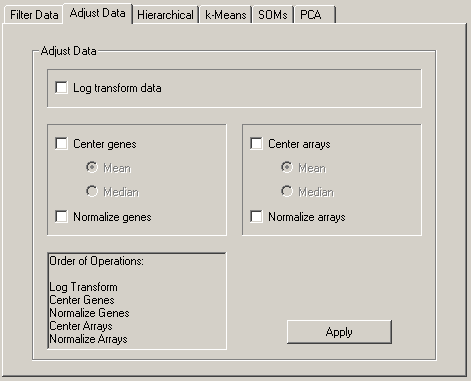

Supplement: Supplementary file 12 — Dataset EV8 [file MSB-11-842-s016.zip › Dataset_EV8/Cluster.app/Contents/Resources/html/images/adjust.png]

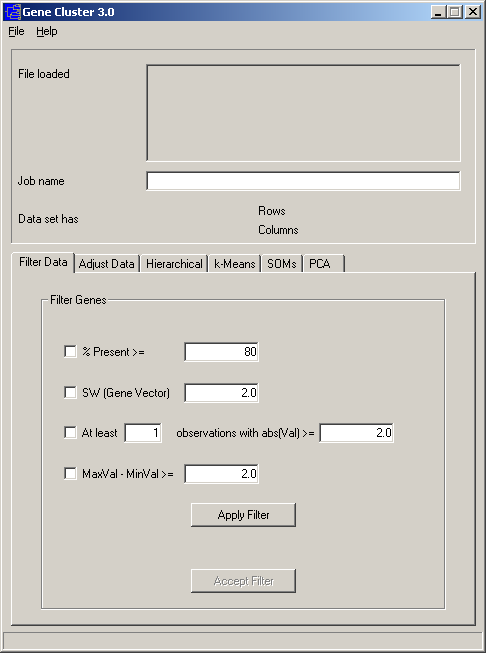

Supplement: Supplementary file 12 — Dataset EV8 [file MSB-11-842-s016.zip › Dataset_EV8/Cluster.app/Contents/Resources/html/images/cluster.png]

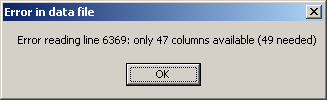

Supplement: Supplementary file 12 — Dataset EV8 [file MSB-11-842-s016.zip › Dataset_EV8/Cluster.app/Contents/Resources/html/images/fileerror.png]

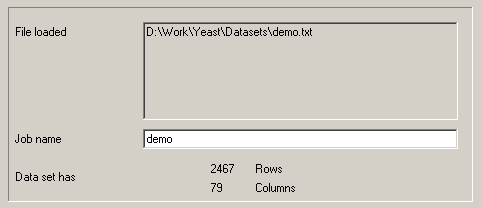

Supplement: Supplementary file 12 — Dataset EV8 [file MSB-11-842-s016.zip › Dataset_EV8/Cluster.app/Contents/Resources/html/images/filemanager.png]

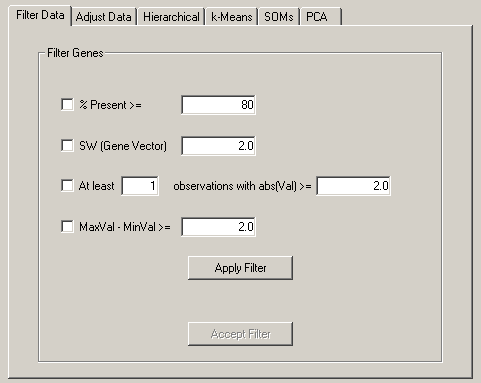

Supplement: Supplementary file 12 — Dataset EV8 [file MSB-11-842-s016.zip › Dataset_EV8/Cluster.app/Contents/Resources/html/images/filter.png]

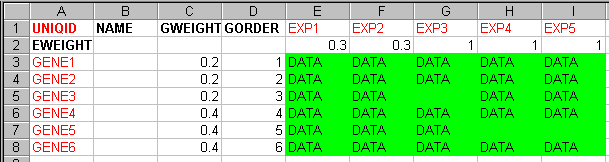

Supplement: Supplementary file 12 — Dataset EV8 [file MSB-11-842-s016.zip › Dataset_EV8/Cluster.app/Contents/Resources/html/images/format.png]

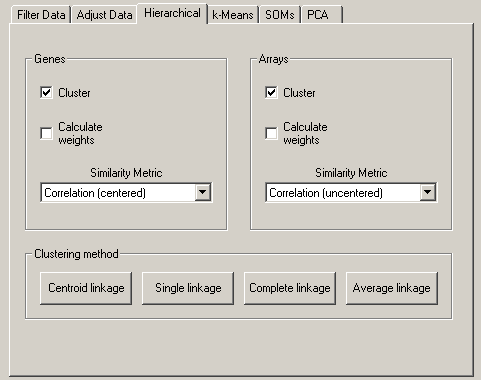

Supplement: Supplementary file 12 — Dataset EV8 [file MSB-11-842-s016.zip › Dataset_EV8/Cluster.app/Contents/Resources/html/images/hierarchical.png]

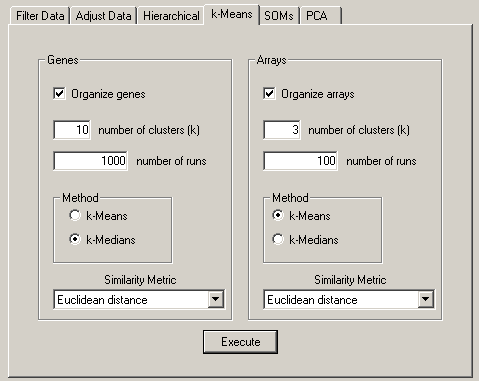

Supplement: Supplementary file 12 — Dataset EV8 [file MSB-11-842-s016.zip › Dataset_EV8/Cluster.app/Contents/Resources/html/images/kmeans.png]

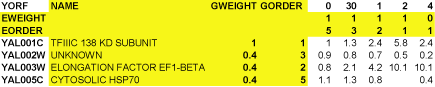

Supplement: Supplementary file 12 — Dataset EV8 [file MSB-11-842-s016.zip › Dataset_EV8/Cluster.app/Contents/Resources/html/images/maxifile.png]

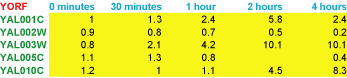

Supplement: Supplementary file 12 — Dataset EV8 [file MSB-11-842-s016.zip › Dataset_EV8/Cluster.app/Contents/Resources/html/images/minifile.png]

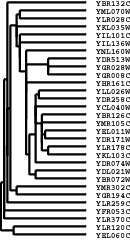

Supplement: Supplementary file 12 — Dataset EV8 [file MSB-11-842-s016.zip › Dataset_EV8/Cluster.app/Contents/Resources/html/images/order.png]

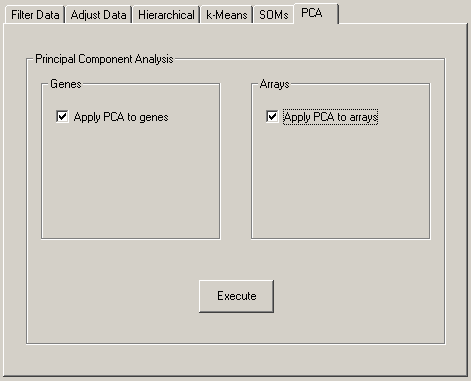

Supplement: Supplementary file 12 — Dataset EV8 [file MSB-11-842-s016.zip › Dataset_EV8/Cluster.app/Contents/Resources/html/images/pca.png]

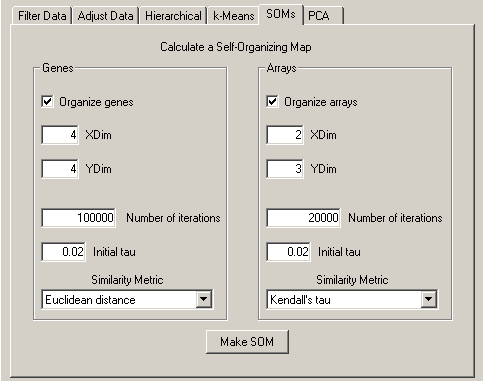

Supplement: Supplementary file 12 — Dataset EV8 [file MSB-11-842-s016.zip › Dataset_EV8/Cluster.app/Contents/Resources/html/images/som.png]

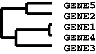

Supplement: Supplementary file 12 — Dataset EV8 [file MSB-11-842-s016.zip › Dataset_EV8/Cluster.app/Contents/Resources/html/images/tree.png]

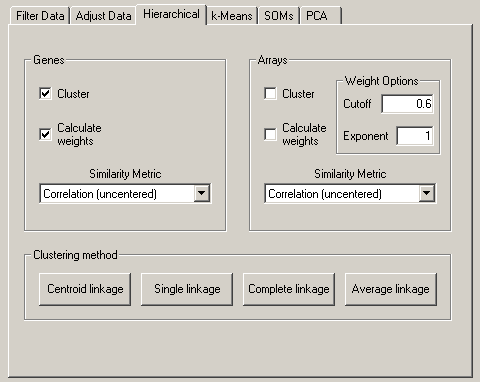

Supplement: Supplementary file 12 — Dataset EV8 [file MSB-11-842-s016.zip › Dataset_EV8/Cluster.app/Contents/Resources/html/images/weight.png]

**Supplementary Dataset 8**

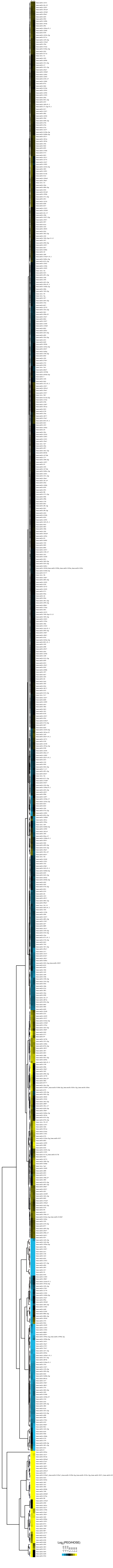

Supplement: Supplementary file 12 — Dataset EV8 [file MSB-11-842-s016.zip › Dataset_EV8/Dataset EV8.pdf]

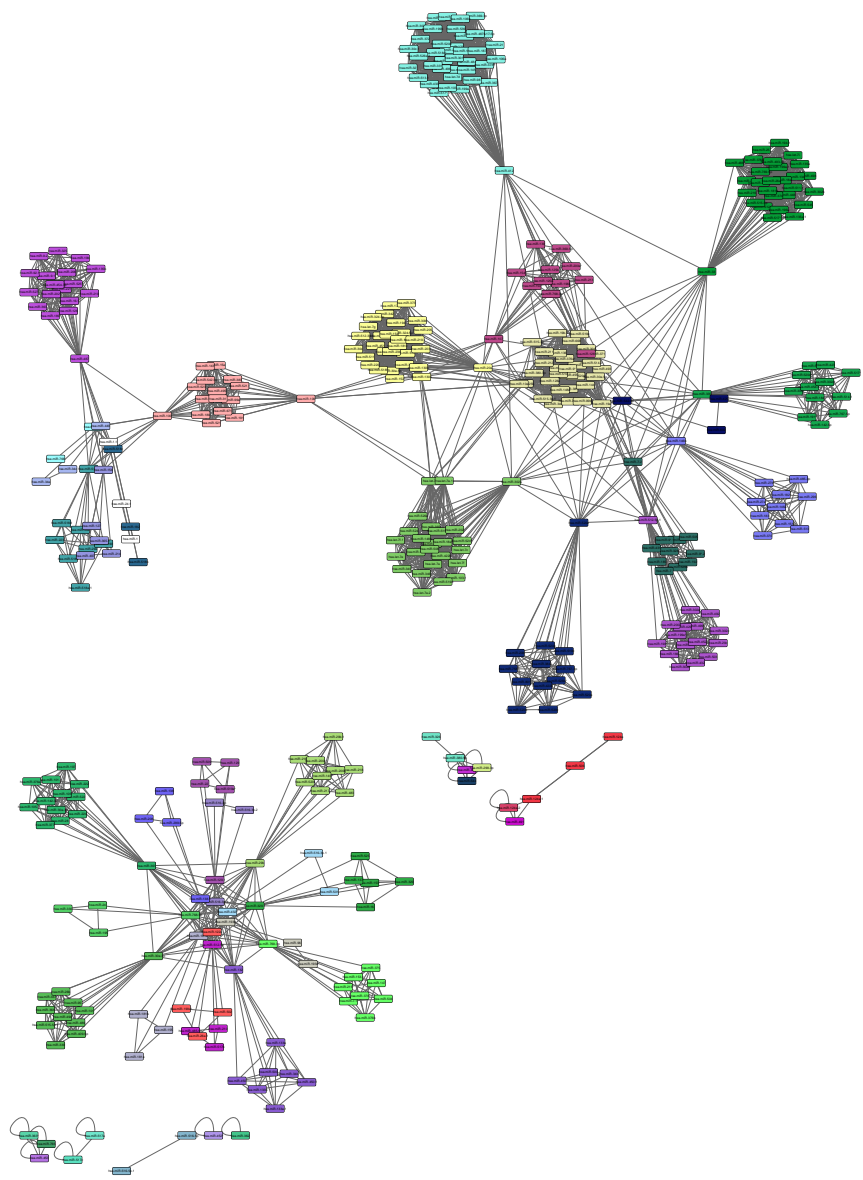

Supplement: Supplementary file 15 — Dataset EV11 [file MSB-11-842-s019.zip › Dataset_EV11/Dataset EV11.pdf]

# Uncut Blots for Figure 2B

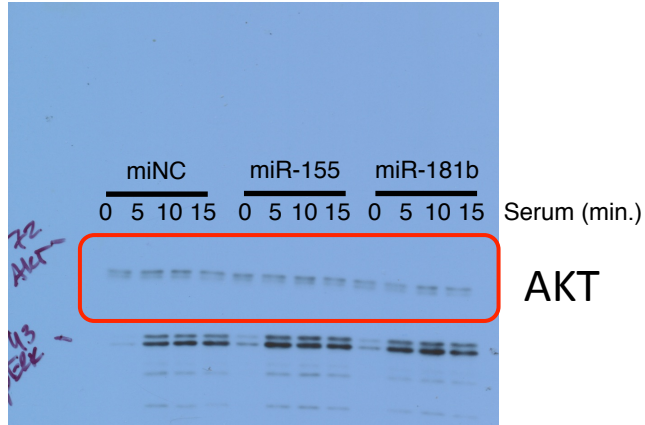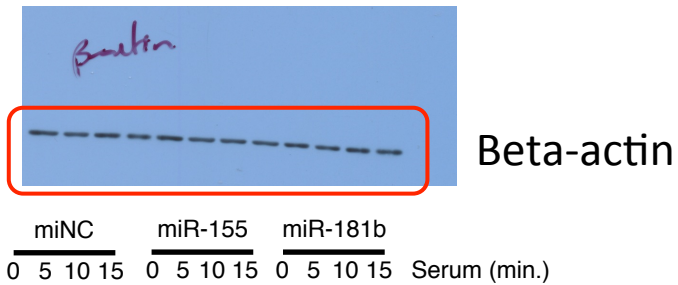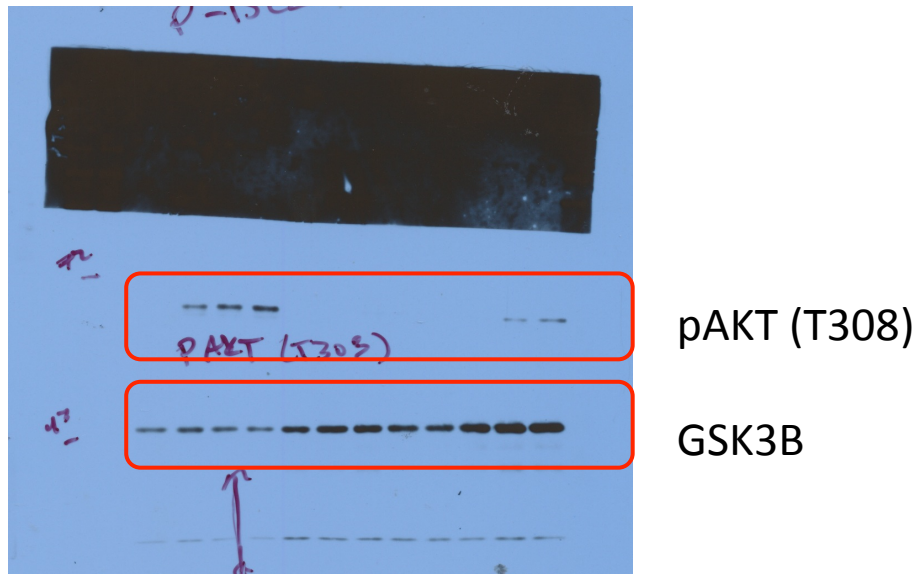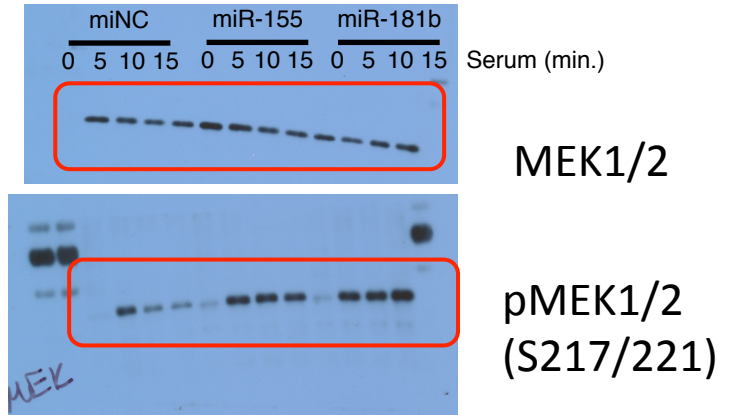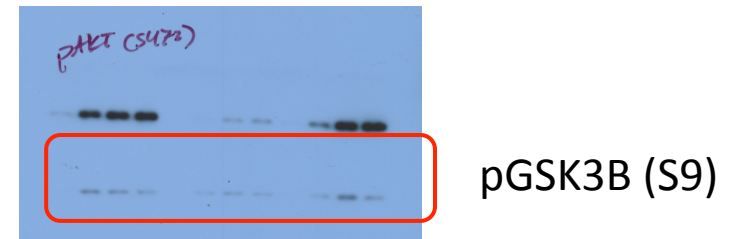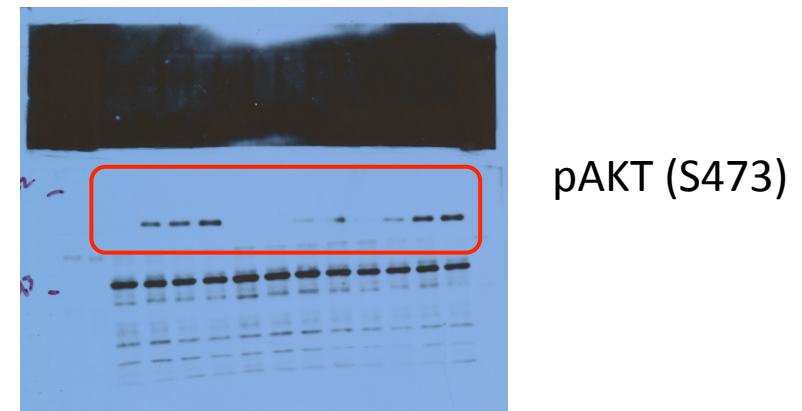

Lanes used highlighted in red

Supplement: Supplementary file 17 — Source Data for Figure 2 [file MSB-11-842-s005.zip › Source Data Fig 2/Source Data Figure 2B.pdf]

# Uncut Blots for Figure 3E

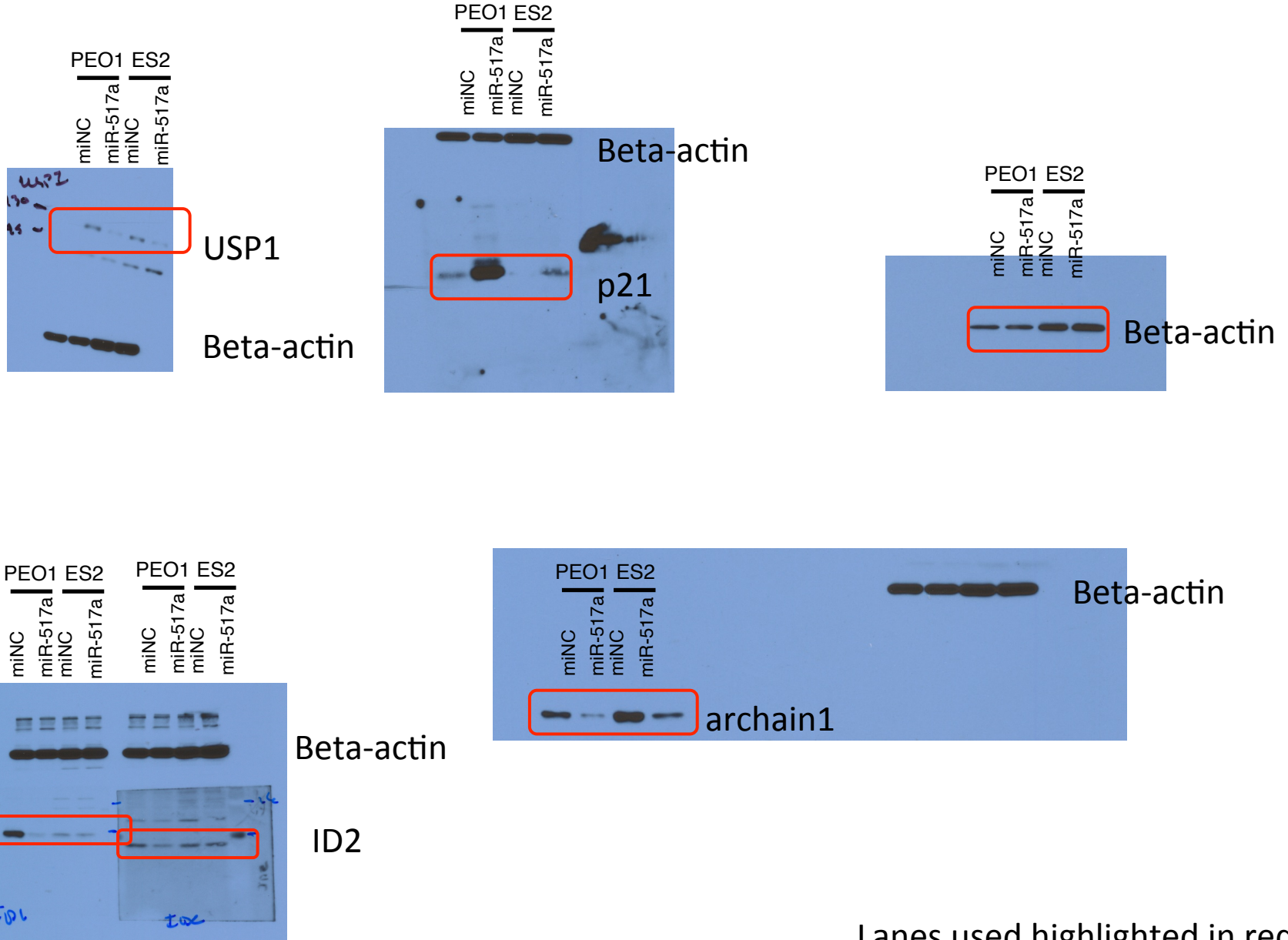

Supplement: Supplementary file 18 — Source Data for Figure 3 [file MSB-11-842-s006.zip › Source Data Fig 3/Source Data Figure 3E.pdf]

# Uncut Blot for Figure 5A

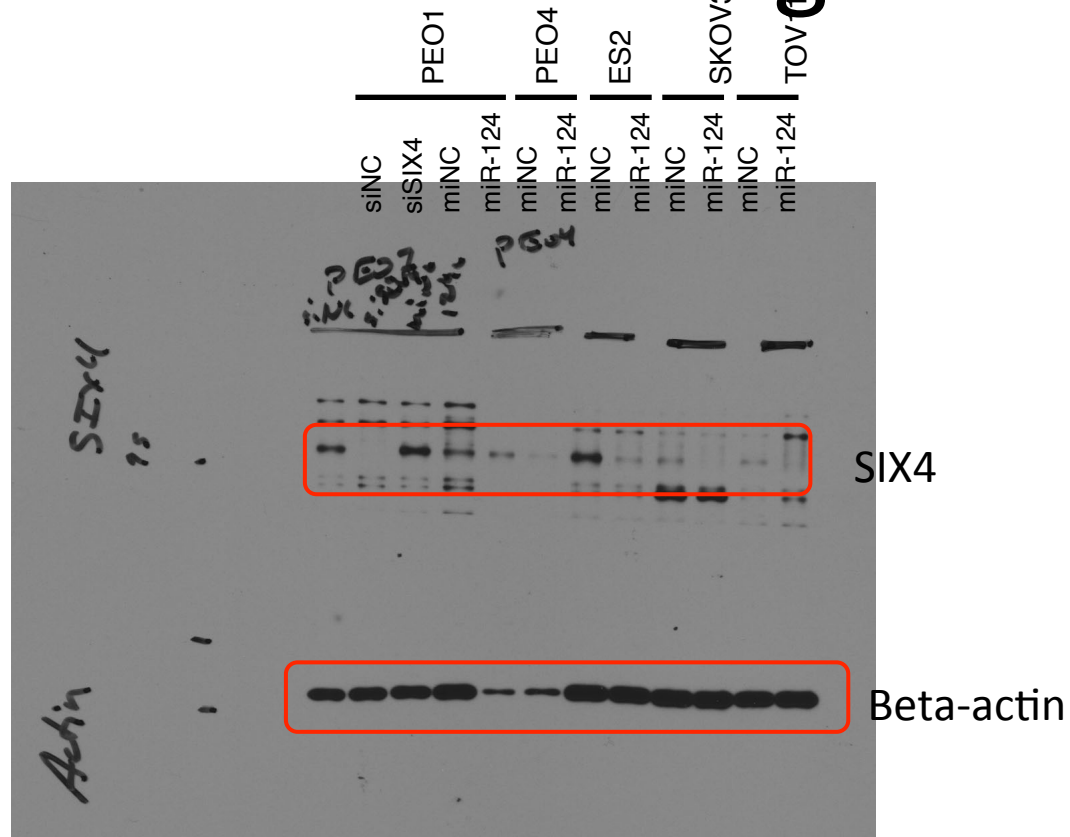

Lanes used highlighted in red

Supplement: Supplementary file 20 — Source Data for Figure 5 [file MSB-11-842-s008.zip › Source Data Fig 5/Source Data Figure 5A.pdf]

# Uncut Blot for Figure 5E

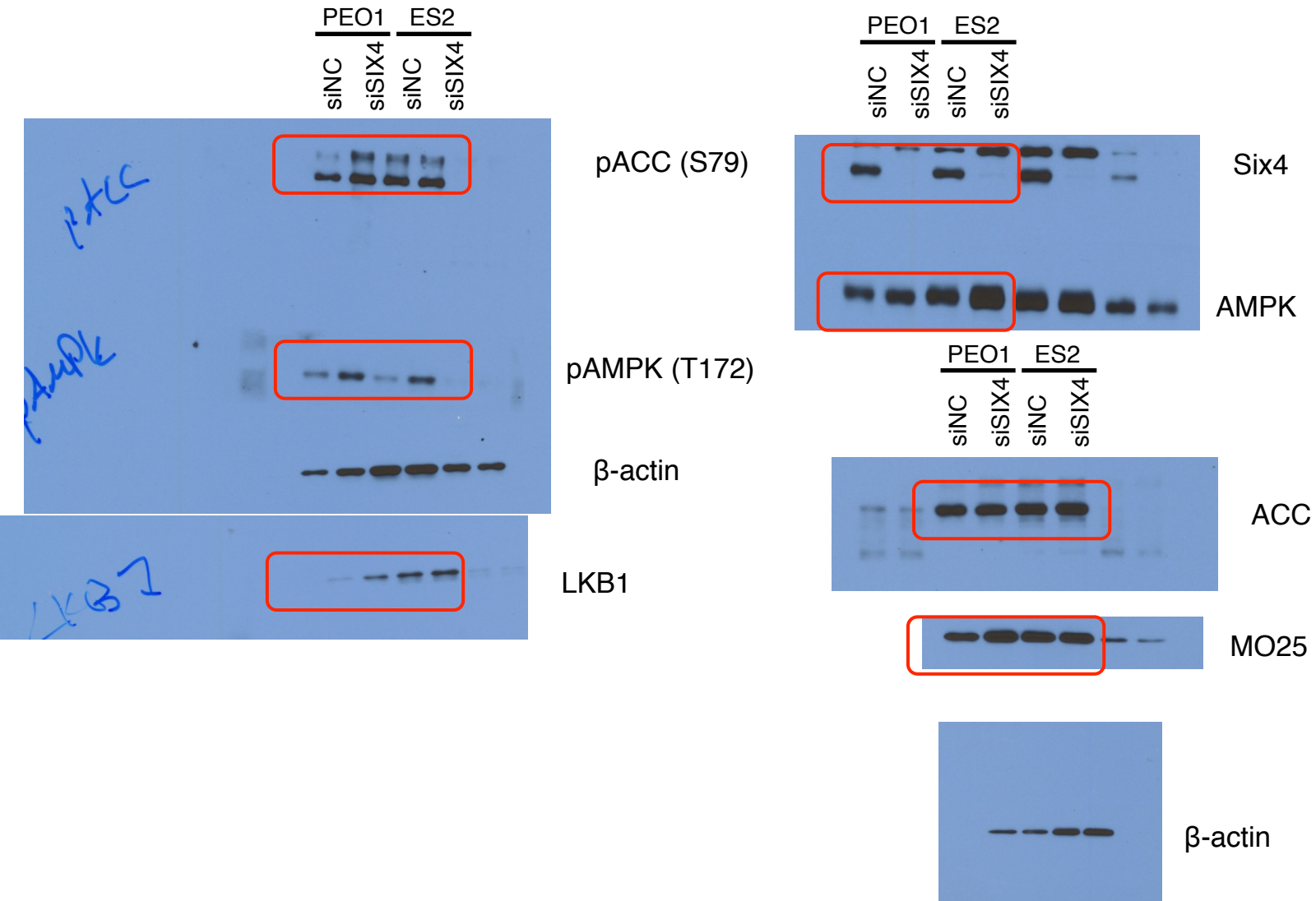

Lanes used highlighted in red

Supplement: Supplementary file 20 — Source Data for Figure 5 [file MSB-11-842-s008.zip › Source Data Fig 5/Source Data Figure 5E.pdf]
