# Supplementary material for: A genome‐scale screen reveals context‐dependent ovarian cancer sensitivity to miRNA overexpression
Source: Mol Syst Biol. 2015 Dec 11;11(12):842. doi: 10.15252/msb.20156308 (PMC4704493; doi:10.15252/msb.20156308)
Supplement: Supplementary file 12 — Dataset EV8 [file MSB-11-842-s016.zip › Dataset_EV8/Cluster.app/Contents/Resources/cluster3.pdf]

Cluster 3.0 Manual

for Windows, Mac OS X, Linux, Unix

Michael Eisen; updated by Michiel de Hoon

Software copyright © Stanford University 1998-99 This manual was originally written by Michael Eisen. It is only partially complete and is a work in progress. The manual was updated in 2002 by Michiel de Hoon, University of Tokyo, Human Genome Center.

This is the manual for Cluster 3.0. Cluster was originally written by Michael Eisen while at Stanford University. We have modified the k-means clustering algorithm in Cluster, and extended the algorithm for Self-Organizing Maps to include two-dimensional rectangular grids. The Euclidean distance and the city-block distance were added as new distance measures between gene expression data. The proprietary Numerical Recipes routines, which were used in the original version of Cluster/TreeView, have been replaced by open source software.

Cluster 3.0 is available for Windows, Mac OS X, Linux, and Unix.

November 5, 2002.
Michiel de Hoon
Human Genome Center, University of Tokyo.

Table of Contents

1 Introduction 2

2 Loading, filtering, and adjusting data 3
- 2.1 Loading Data 4
- 2.2 Filtering Data 6
- 2.3 Adjusting Data 7
  - 2.3.1 Log transformation 8
  - 2.3.2 Mean/Median Centering 8
  - 2.3.3 Normalization 9

3 Distance/Similarity measures 10
- 3.1 Distance measures based on the Pearson correlation 10
- 3.2 Non-parametric distance measures 11
- 3.3 Distance measures related to the Euclidean distance 12
  - 3.3.1 Euclidean distance 12
  - 3.3.2 City-block distance 12
- 3.4 Missing values 12
- 3.5 Calculating the distance matrix 13

4 Clustering techniques 14
- 4.1 Hierarchical Clustering 14
  - 4.1.1 Centroid Linkage Clustering 15
  - 4.1.2 Single Linkage Clustering 16
  - 4.1.3 Complete Linkage Clustering 16
  - 4.1.4 Average Linkage Clustering 16
  - 4.1.5 Weighting 16
  - 4.1.6 Ordering of Output File 17
  - 4.1.7 Output Files 18
- 4.2 The \math k-means Clustering Algorithm 20
- 4.3 Self-Organizing Maps 22
- 4.4 Principal Component Analysis 23

5 Running Cluster 3.0 as a command line program 26

6 TreeView 28

7 Code Development Information 29

8 Bibliography 30

1 Introduction

Cluster and TreeView are programs that provide a computational and graphical environment for analyzing data from DNA microarray experiments, or other genomic datasets. The program Cluster can organize and analyze the data in a number of different ways. TreeView allows the organized data to be visualized and browsed.

This manual is intended as a reference for using the software, and not as a comprehensive introduction to the methods employed. Many of the methods are drawn from standard statistical cluster analysis. There are excellent textbooks available on cluster analysis which are listed in the bibliography at the end. The bibliography also contains citations for recent publications in the biological sciences, especially genomics, that employ methods similar to those used here.

2 Loading, filtering, and adjusting data

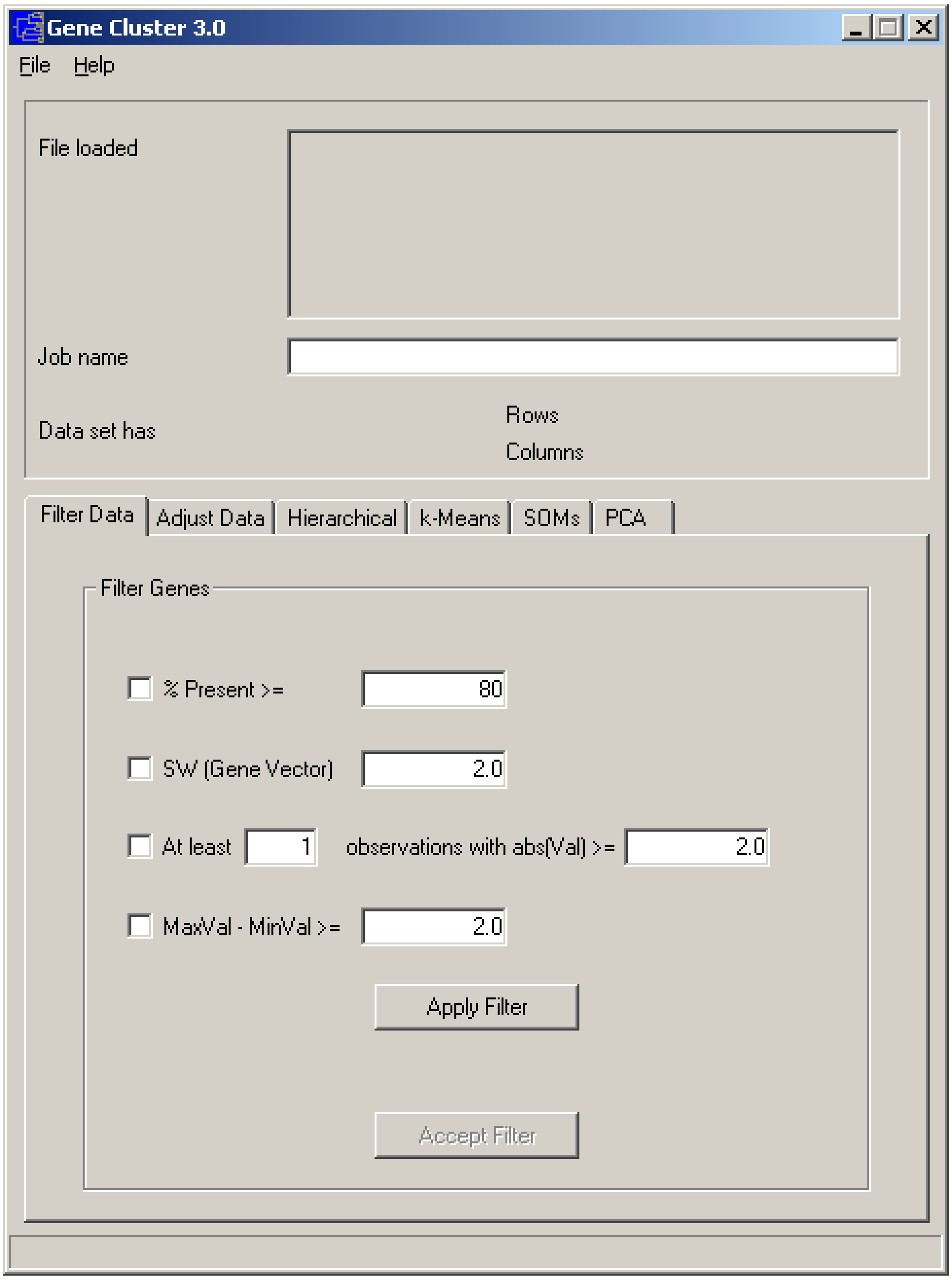

Data can be loaded into Cluster by choosing Load data file under the File menu. A number of options are provided for adjusting and filtering the data you have loaded. These functions are accessed via the Filter Data and Adjust Data tabs.

2.1 Loading Data

The first step in using Cluster is to import data. Currently, Cluster only reads tab-delimited text files in a particular format, described below. Such tab-delimited text files can be created and exported in any standard spreadsheet program, such as Microsoft Excel. An example datafile can be found under the File format help item in the Help menu. This contains all the information you need for making a Cluster input file.

By convention, in Cluster input tables rows represent genes and columns represent samples or observations (e.g. a single microarray hybridization). For a simple timecourse, a minimal Cluster input file would look like this:

YORF	0 minutes	30 minutes	1 hour	2 hours	4 hours
YAL001C	1	1.3	2.4	5.8	2.4
YAL002W	0.9	0.8	0.7	0.5	0.2
YAL003W	0.8	2.1	4.2	10.1	10.1
YAL005C	1.1	1.3	0.8		0.4
YAL010C	1.2	1	1.1	4.5	8.3

Each row (gene) has an identifier (in green) that always goes in the first column. Here we are using yeast open reading frame codes. Each column (sample) has a label (in blue) that is always in the first row; here the labels describe the time at which a sample was taken. The first column of the first row contains a special field (in red) that tells the program what kind of objects are in each row. In this case, YORF stands for yeast open reading frame. This field can be any alpha-numeric value. It is used in TreeView to specify how rows are linked to external websites.

The remaining cells in the table contain data for the appropriate gene and sample. The 5.8 in row 2 column 4 means that the observed data value for gene YAL001C at 2 hours was 5.8. Missing values are acceptable and are designated by empty cells (e.g. YAL005C at 2 hours).

It is possible to have additional information in the input file. A maximal Cluster input file would look like this:

YORF	NAME	GWEIGHT	GORDER	0	30	1	2	4
EWEIGHT				1	1	1	1	0
EORDER				5	3	2	1	1
YAL001C	TFIIIC 138 KD SUBUNIT	1	1	1	1.3	2.4	5.8	2.4
YAL002W	UNKNOWN	0.4	3	0.9	0.8	0.7	0.5	0.2
YAL003W	ELONGATION FACTOR EF1-BETA	0.4	2	0.8	2.1	4.2	10.1	10.1
YAL005C	CYTOSOLIC HSP70	0.4	5	1.1	1.3	0.8		0.4

The yellow columns and rows are optional. By default, TreeView uses the ID in column 1 as a label for each gene. The NAME column allows you to specify a label for each gene that is distinct from the ID in column 1. The other rows and columns will be described later in this text.

When Cluster 3.0 opens the data file, the number of columns in each row is checked. If a given row contains less or more columns than needed, an error message is displayed.

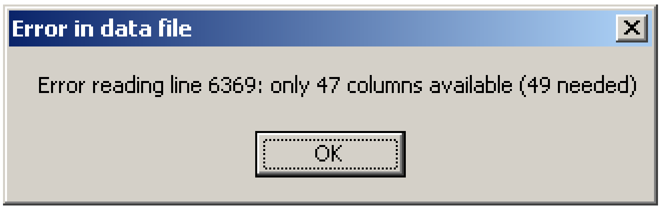

Demo data

A demo datafile, which will be used in all of the examples here, is available at `http://rana.lbl.gov/downloads/data/demo.txt` and is mirrored at `http://bonsai.ims.u-tokyo.ac.jp/~mdehoon/software/cluster/demo.txt`.

The datafile contains yeast gene expression data described in Eisen *et al.* (1998) [see references at end]. Download this data and load it into Cluster. Cluster will give you information about the loaded datafile.

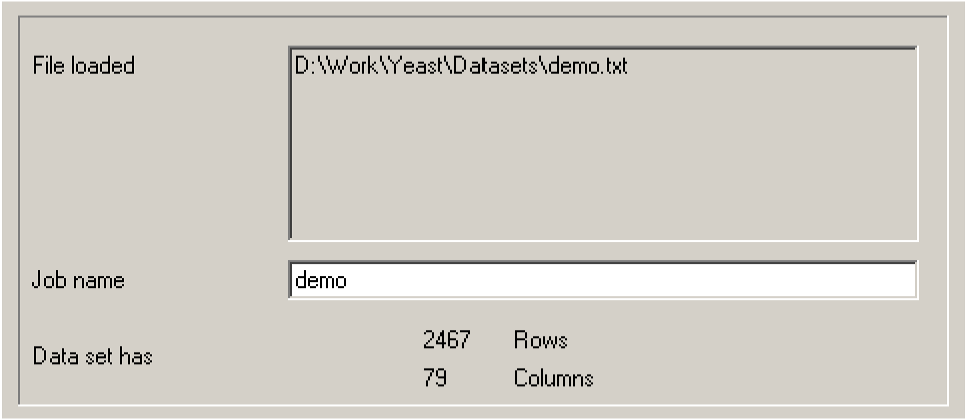

2.2 Filtering Data

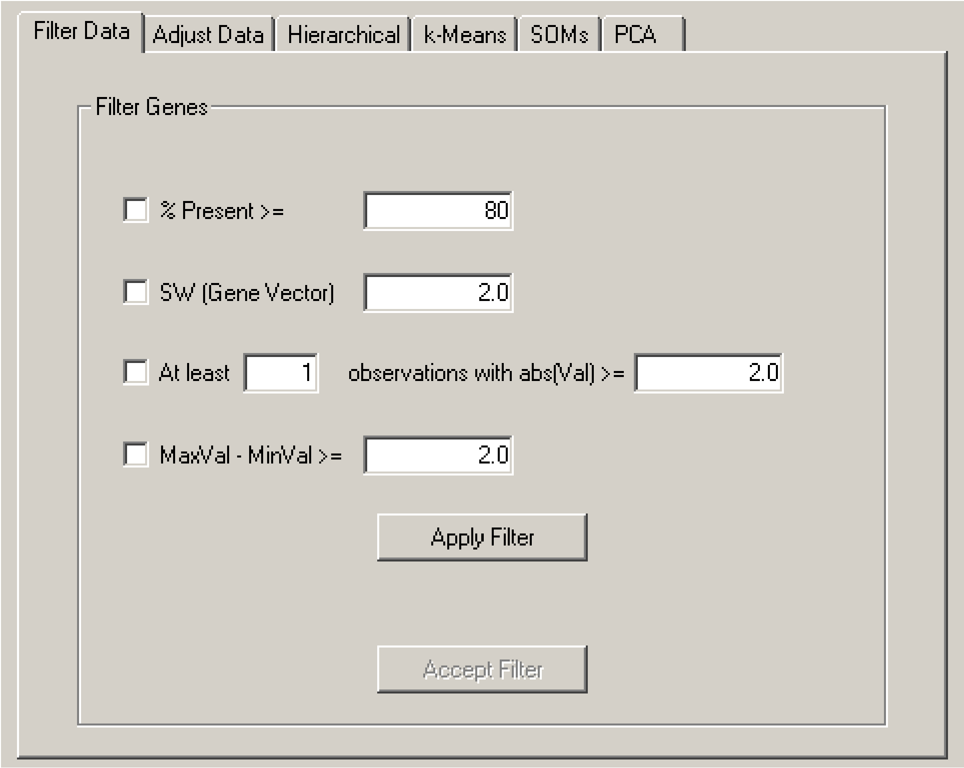

The Filter Data tab allows you to remove genes that do not have certain desired properties from your dataset. The currently available properties that can be used to filter data are

- **% Present >= X**. This removes all genes that have missing values in greater than $(100 - X)$ percent of the columns.
- **SD (Gene Vector) >= X**. This removes all genes that have standard deviations of observed values less than X.
- **At least X Observations with abs(Val) >= Y**. This removes all genes that do not have at least X observations with absolute values greater than Y.
- **MaxVal-MinVal >= X**. This removes all genes whose maximum minus minimum values are less than X.

These are fairly self-explanatory. When you press filter, the filters are not immediately applied to the dataset. You are first told how many genes would have passed the filter. If

you want to accept the filter, you press Accept, otherwise no changes are made.

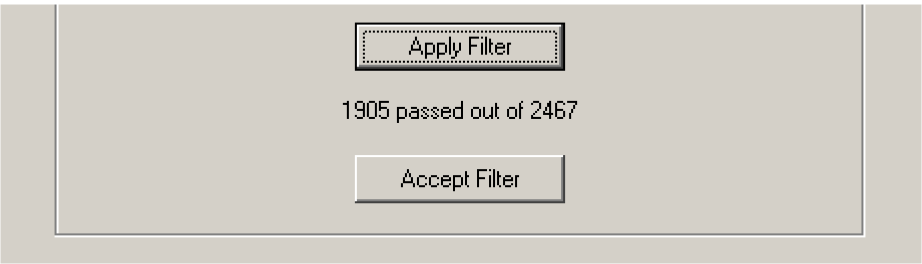

2.3 Adjusting Data

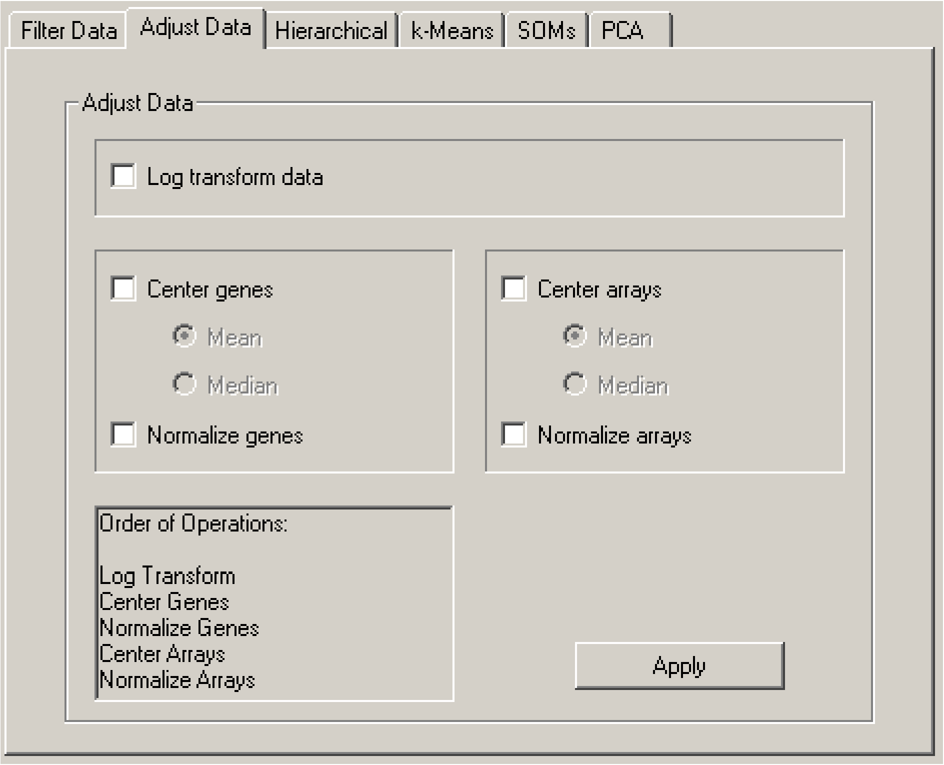

From the Adjust Data tab, you can perform a number of operations that alter the underlying data in the imported table. These operations are

- **Log Transform Data**: replace all data values x by $\log_2(x)$.
- **Center genes [mean or median]**: Subtract the row-wise mean or median from the values in each row of data, so that the mean or median value of each row is 0.
- **Center arrays [mean or median]**: Subtract the column-wise mean or median from the values in each column of data, so that the mean or median value of each column is 0.
- **Normalize genes**: Multiply all values in each row of data by a scale factor S so that the sum of the squares of the values in each row is 1.0 (a separate S is computed for each row).

- **Normalize arrays**: Multiply all values in each column of data by a scale factor S so that the sum of the squares of the values in each column is 1.0 (a separate S is computed for each column).

These operations are not associative, so the order in which these operations is applied is very important, and you should consider it carefully before you apply these operations. The order of operations is (only checked operations are performed):

- Log transform all values.
- Center rows by subtracting the mean or median.
- Normalize rows.
- Center columns by subtracting the mean or median.
- Normalize columns.

2.3.1 Log transformation

The results of many DNA microarray experiments are fluorescent ratios. Ratio measurements are most naturally processed in log space. Consider an experiment where you are looking at gene expression over time, and the results are relative expression levels compared to time 0. Assume at timepoint 1, a gene is unchanged, at timepoint 2 it is up 2-fold and at timepoint three is down 2-fold relative to time 0. The raw ratio values are 1.0, 2.0 and 0.5. In most applications, you want to think of 2-fold up and 2-fold down as being the same magnitude of change, but in an opposite direction. In raw ratio space, however, the difference between timepoint 1 and 2 is +1.0, while between timepoint 1 and 3 is -0.5. Thus mathematical operations that use the difference between values would think that the 2-fold up change was twice as significant as the 2-fold down change. Usually, you do not want this. In log space (we use log base 2 for simplicity) the data points become 0,1.0,-1.0.With these values, 2-fold up and 2-fold down are symmetric about 0. For most applications, we recommend you work in log space.

2.3.2 Mean/Median Centering

Consider a now common experimental design where you are looking at a large number of tumor samples all compared to a common reference sample made from a collection of cell-lines. For each gene, you have a series of ratio values that are relative to the expression level of that gene in the reference sample. Since the reference sample really has nothing to do with your experiment, you want your analysis to be independent of the amount of a gene present in the reference sample. This is achieved by adjusting the values of each gene to reflect their variation from some property of the series of observed values such as the mean or median. This is what mean and/or median centering of genes does. Centering makes less sense in experiments where the reference sample is part of the experiment, as it is many timecourses. Centering the data for columns/arrays can also be used to remove certain types of biases. The results of many two-color fluorescent hybridization experiments are not corrected for systematic biases in ratios that are the result of differences in RNA amounts, labeling efficiency and image acquisition parameters. Such biases have the effect of multiplying ratios for all genes by a fixed scalar. Mean or median centering the data in log-space has the effect of correcting this bias, although it should be noted that an assumption is being made in correcting this bias, which is that the average gene in a given experiment is expected to have a ratio of 1.0 (or log-ratio of 0).

In general, I recommend the use of median rather than mean centering, as it is more robust against outliers.

2.3.3 Normalization

Normalization sets the magnitude (sum of the squares of the values) of a row/column vector to 1.0. Most of the distance metrics used by Cluster work with internally normalized data vectors, but the data are output as they were originally entered. If you want to output normalized vectors, you should select this option. A sample series of operations for raw data would be:

- Adjust Cycle 1) log transform
- Adjust Cycle 2) median center genes and arrays
- repeat (2) five to ten times
- Adjust Cycle 3) normalize genes and arrays
- repeat (3) five to ten times

This results in a log-transformed, median polished (i.e. all row-wise and column-wise median values are close to zero) and normal (i.e. all row and column magnitudes are close to 1.0) dataset. After performing these operations you should save the dataset.

3 Distance/Similarity measures

The first choice that must be made is how similarity (or alternatively, distance) between gene expression data is to be defined. There are many ways to compute how similar two series of numbers are. Cluster provides eight options.

3.1 Distance measures based on the Pearson correlation

The most commonly used similarity metrics are based on Pearson correlation. The Pearson correlation coefficient between any two series of numbers $x = \{x_1, x_2, \ldots, x_n\}$ and $y = \{y_1, y_2, \ldots, y_n\}$ is defined as

$$r = \frac{1}{n} \sum_{i=1}^{n} \left(\frac{x_i - \overline{x}}{\sigma_x} \right) \left(\frac{y_i - \overline{y}}{\sigma_y} \right),$$

where $\overline{x}$ is the average of values in x, and σ_x is the standard deviation of these values.

There are many ways of conceptualizing the correlation coefficient. If you were to make a scatterplot of the values of x against y (pairing x_1 with y_1, x_2 with y_2, etc), then r reports how well you can fit a line to the values.

The simplest way to think about the correlation coefficient is to plot x and y as curves, with r telling you how similar the shapes of the two curves are. The Pearson correlation coefficient is always between -1 and 1, with 1 meaning that the two series are identical, 0 meaning they are completely uncorrelated, and -1 meaning they are perfect opposites. The correlation coefficient is invariant under linear transformation of the data. That is, if you multiply all the values in y by 2, or add 7 to all the values in y, the correlation between x and y will be unchanged. Thus, two curves that have identical shape, but different magnitude, will still have a correlation of 1.

Cluster actually uses four different flavors of the Pearson correlation. The textbook Pearson correlation coefficient, given by the formula above, is used if you select Correlation (centered) in the Similarity Metric dialog box. Correlation (uncentered) uses the following modified equations:

$$r = \frac{1}{n} \sum_{i=1}^{n} \left(\frac{x_i}{\sigma_x^{(0)}} \right) \left(\frac{y_i}{\sigma_y^{(0)}} \right),$$

in which

$$\sigma_x^{(0)} = \sqrt{\frac{1}{n} \sum_{i=1}^{n} (x_i)^2};$$

$$\sigma_y^{(0)} = \sqrt{\frac{1}{n} \sum_{i=1}^{n} (y_i)^2}.$$

This is basically the same function, except that it assumes the mean is 0, even when it is not. The difference is that, if you have two vectors x and y with identical shape, but which are offset relative to each other by a fixed value, they will have a standard Pearson

correlation (centered correlation) of 1 but will not have an uncentered correlation of 1. The uncentered correlation is equal to the cosine of the angle of two n-dimensional vectors x and y, each representing a vector in n-dimensional space that passes through the origin. Cluster provides two similarity metrics that are the absolute value of these two correlation functions, which consider two items to be similar if they have opposite expression patterns; the standard correlation coefficients consider opposite genes to be very distant.

3.2 Non-parametric distance measures

The Spearman rank correlation and Kendall's τ are two additional metrics, which are non-parametric versions of the Pearson correlation coefficient. These methods are more robust against outliers.

The Spearman rank correlation calculates the correlation between the ranks of the data values in the two vectors. For example, if we have two data vectors

$$x = \{2.3, 6.7, 4.5, 20.8\} ;$$
$$y = \{2.1, 5.9, 4.4, 4.2\} ,$$

then we first replace them by their ranks:

$$x = \{1, 3, 2, 4\} ;$$
$$y = \{1, 4, 3, 2\} .$$

Now we calculate the correlation coefficient in their usual manner from these data vectors, resulting in

$$r_{\text{Spearman}} = 0.4.$$

In comparison, the regular Pearson correlation between these data is $r = 0.2344$. By replacing the data values by their ranks, we reduced the effect of the outlier 20.8 on the value of the correlation coefficient. The Spearman rank correlation can be used as a test statistic for independence between x and y. For more information, see Conover (1980).

Kendall's τ goes a step further by using only the relative ordering of x and y to calculate the correlation (Snedecor & Cochran). To calculate Kendall's τ, consider all pairs of data points (x_i, y_i) and (x_j, y_j). We call a pair concordant if

- $x_i < x_j$ and $y_i < y_j$; or
- $x_i > x_j$ and $y_i > y_j$,

and discordant if

- $x_i < x_j$ and $y_i > y_j$; or
- $x_i > x_j$ and $y_i < y_j$.

We can represent this by a table:

–	(2.3, 2.1)	(6.7, 5.9)	(4.5, 4.4)	(20.8, 4.2)
(2.3, 2.1)	–	$<<$	$<<$	$<<$
(6.7, 5.9)	$>>$	–	$>>$	$<>$
(4.5, 4.4)	$>>$	$<<$	–	$<>$
(20.8, 4.2)	$>>$	$><$	$><$	–

From this table, we find that there are four concordant pairs and two discordant pairs:

$$n_{\mathrm{c}} = 4;$$

$$n_{\mathrm{d}} = 2;$$

Kendall's τ is calculated as

$$\frac{\tau = N_{\mathrm{c}} - N_{\mathrm{d}}}{N\left(N-1\right)/2,}$$

which in this case evaluates as 0.33. In the C Clustering Library, the calculation of Kendall's τ is corrected for the possibility that two ranks are equal. As in case of the Spearman rank correlation, we may use Kendall's τ to test for independence between x and y.

3.3 Distance measures related to the Euclidean distance

3.3.1 Euclidean distance

A newly added distance function is the Euclidean distance, which is defined as

$$d\left(\underline{x}, \underline{y}\right) = \frac{1}{n} \sum_{i=1}^{n} \left(x_i - y_i\right)^2 .$$

The Euclidean distance takes the difference between two gene expression levels directly. It should therefore only be used for expression data that are suitably normalized, for example by converting the measured gene expression levels to log-ratios. In the sum, we only include terms for which both x_i and y_i are present, and divide by n accordingly.

Unlike the correlation-based distance measures, the Euclidean distance takes the magnitude of changes in the gene expression levels into account. An example of the Euclidean distance applied to k-means clustering can be found in De Hoon, Imoto, and Miyano (2002).

3.3.2 City-block distance

The city-block distance, alternatively known as the Manhattan distance, is related to the Euclidean distance. Whereas the Euclidean distance corresponds to the length of the shortest path between two points, the city-block distance is the sum of distances along each dimension:

$$d = \sum_{i=1}^{n} \left|x_i - y_i\right| .$$

This is equal to the distance you would have to walk between two points in a city, where you have to walk along city blocks. The city-block distance is a metric, as it satisfies the triangle inequality. Again we only include terms for which both x_i and y_i are present, and divide by n accordingly.

As for the Euclidean distance, the expression data are subtracted directly from each other, and we should therefore make sure that they are properly normalized.

3.4 Missing values

When either x or y has missing values, only observations present for both x and y are used in computing similarities.

3.5 Calculating the distance matrix

With any specified metric, the first step in the hierarchical clustering routines described below is to compute the distance (the opposite of similarity; for all correlation metrics distance = 1.0 - correlation) between all pairs of items to be clustered (e.g. the set of genes in the current dataset). This can often be time consuming, and, except for pairwise single-linkage clustering, memory intensive (the maximum amount of memory required is $4 \times N \times N$ bytes, where N is the number of items being clustered). The algorithm for pairwise single-linkage hierarchical clustering is less memory-intensive (linear in N).

4 Clustering techniques

The Cluster program provides several clustering algorithms. Hierarchical clustering methods organizes genes in a tree structure, based on their similarity. Four variants of hierarchical clustering are available in Cluster. In k-means clustering, genes are organized into k clusters, where the number of clusters k needs to be chosen in advance. Self-Organizing Maps create clusters of genes on a two-dimensional rectangular grid, where neighboring clusters are similar. For each of these methods, one of the eight different distance meaures can be used. Finally, in Principal Component Analysis, clusters are organized based on the principal component axes of the distance matrix.

4.1 Hierarchical Clustering

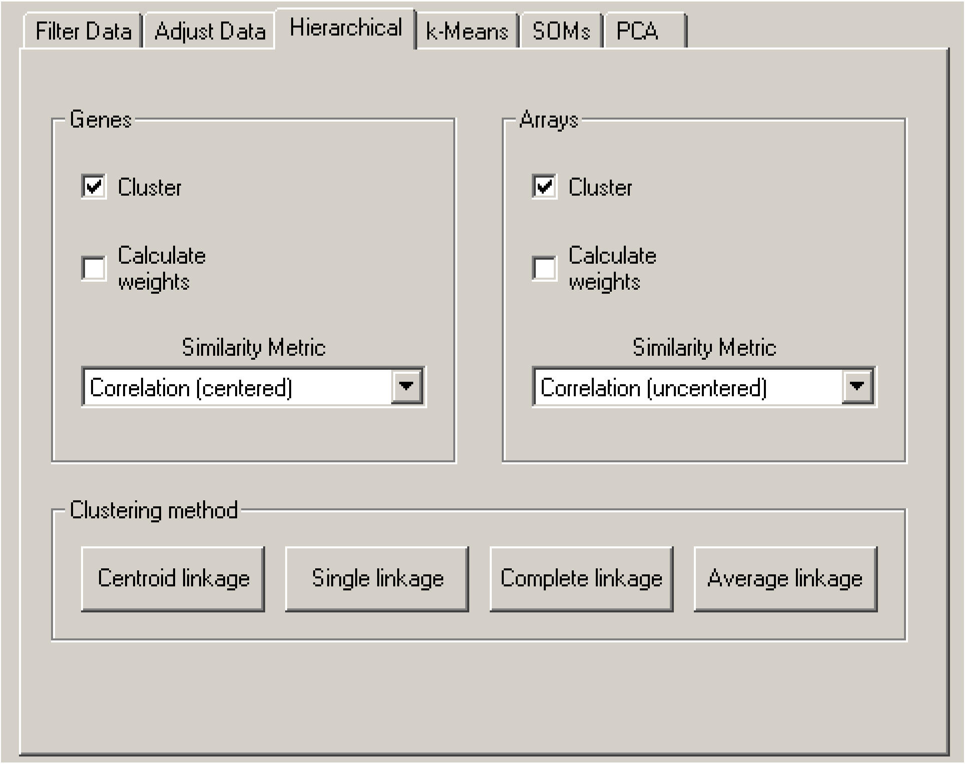

The *Hierarchical Clustering* tab allows you to perform hierarchical clustering on your data. This is a powerful and useful method for analyzing all sorts of large genomic datasets. Many published applications of this analysis are given in the references section at the end.

Cluster currently performs four types of binary, agglomerative, hierarchical clustering. The basic idea is to assemble a set of items (genes or arrays) into a tree, where items are joined by very short branches if they are very similar to each other, and by increasingly longer branches as their similarity decreases.

The first step in hierarchical clustering is to calculate the distance matrix between the gene expression data. Once this matrix of distances is computed, the clustering begins. Agglomerative hierarchical processing consists of repeated cycles where the two closest

remaining items (those with the smallest distance) are joined by a node/branch of a tree, with the length of the branch set to the distance between the joined items. The two joined items are removed from list of items being processed and replaced by a item that represents the new branch. The distances between this new item and all other remaining items are computed, and the process is repeated until only one item remains.

Note that once clustering commences, we are working with items that are true items (e.g. a single gene) and items that are pseudo-items that contain a number of true items. There are a variety of ways to compute distances when we are dealing with pseudo-items, and Cluster currently provides four choices, which are called centroid linkage, single linkage, complete linkage, and average linkage. **Note that in older versions of Cluster, centroid linkage was referred to as average linkage.**

4.1.1 Centroid Linkage Clustering

If you click Centroid Linkage Clustering, a vector is assigned to each pseudo-item, and this vector is used to compute the distances between this pseudo-item and all remaining items or pseudo-items using the same similarity metric as was used to calculate the initial similarity matrix. The vector is the average of the vectors of all actual items (e.g. genes) contained within the pseudo-item. Thus, when a new branch of the tree is formed joining together a branch with 5 items and an actual item, the new pseudo-item is assigned a vector that is the average of the 6 vectors it contains, and not the average of the two joined items (note that missing values are not used in the average, and a pseudo-item can have a missing value if all of the items it contains are missing values in the corresponding row/column).

Note that from a theoretical perspective, Centroid Linkage Clustering is peculiar if it is used in combination with one of the distance measures that are based on the Pearson correlation. For these distance measures, the data vectors are implicitly normalized when calculating the distance (for example, by subtracting the mean and dividing by the standard deviation when calculating the Pearson correlation. However, when two genes are joined and their centroid is calculated by averaging their data vectors, no normalization is applied. This may lead to the surprising result that distances may decrease when we go up in the tree representing the hierarchical clustering result. For example, consider this data set:

	Exp1	Exp2	Exp3	Exp4
Gene1	0.96	0.07	0.97	0.98
Gene2	0.50	0.28	0.29	0.77
Gene3	0.08	0.96	0.51	0.51
Gene4	0.14	0.19	0.41	0.51

Performing pairwise centroid-linkage hierarchical clustering on this data set, using the Pearson distance as the distance measure, produces the clustering result

- Gene 1 joins Gene 2 at distance 0.47
- (Gene 1, Gene 2) joins Gene 4 at distance 0.46
- (Gene 1, Gene 2, Gene 4) joins Gene 3 at distance 1.62

This may result in ill-formed dendrograms. For an example, see the Java TreeView manual. A solution is to use the Euclidean or the city-block distance, or to use one of the other hierarchical clustering routines, which don't suffer from this issue regardless of the distance measure being used.

4.1.2 Single Linkage Clustering

In Single Linkage Clustering the distance between two items x and y is the minimum of all pairwise distances between items contained in x and y. Unlike centroid linkage clustering, in single linkage clustering no further distances need to be calculated once the distance matrix is known.

In Cluster 3.0, as of version 1.29 the implementation of single linkage clustering is based on the SLINK algorithm (see Sibson, 1973). Whereas this algorithm yields the exact same clustering result as conventional single-linkage hierarchical clustering, it is much faster and more memory-efficient (being linear in the memory requirements, compared to quadratic for the conventional algorithm). Hence, single-linkage hierarchical clustering can be used to cluster large gene expression data sets, for which centroid-, complete-, and average-linkage fail due to lack of memory.

4.1.3 Complete Linkage Clustering

In Complete Linkage Clustering the distance between two items x and y is the maximum of all pairwise distances between items contained in x and y. As in single linkage clustering, no other distances need to be calculated once the distance matrix is known.

4.1.4 Average Linkage Clustering

In average linkage clustering, the distance between two items x and y is the mean of all pairwise distances between items contained in x and y.

4.1.5 Weighting

Weighting: By default, all of the observations for a given item are treated equally. In some cases you may want to give some observations more weight than others. For example, if you have duplicate copies of a gene on your array, you might want to downweight each individual copy when computing distances between arrays. You can specify weights using the '`GWEIGHT`' (gene weight) and '`EWEIGHT`' (experiment weight) parameters in the input file. By default all weights are set to 1.0. Thus, the actual formula, with weights included, for the Pearson correlation of $x = \{x_1, x_2, \ldots, x_n\}$ and $y = \{y_1, y_2, \ldots, y_n\}$ with observation weights of $w = \{w_1, w_2, \ldots, w_n\}$ is

$$r = \frac{1}{\sum_{i=1}^{N} w_i} \sum_{i=1}^{N} w_i \left(\frac{X_i - \overline{X}}{\sigma_X} \right) \left(\frac{Y_i - \overline{Y}}{\sigma_Y} \right)$$

Note that when you are clustering rows (genes), you are using column (array) weights. It is possible to compute weights as well based on a not entirely well understood function. If you want to compute weights for clustering genes, select the check box in the Genes panel

of the Hierarchical Clustering tab.

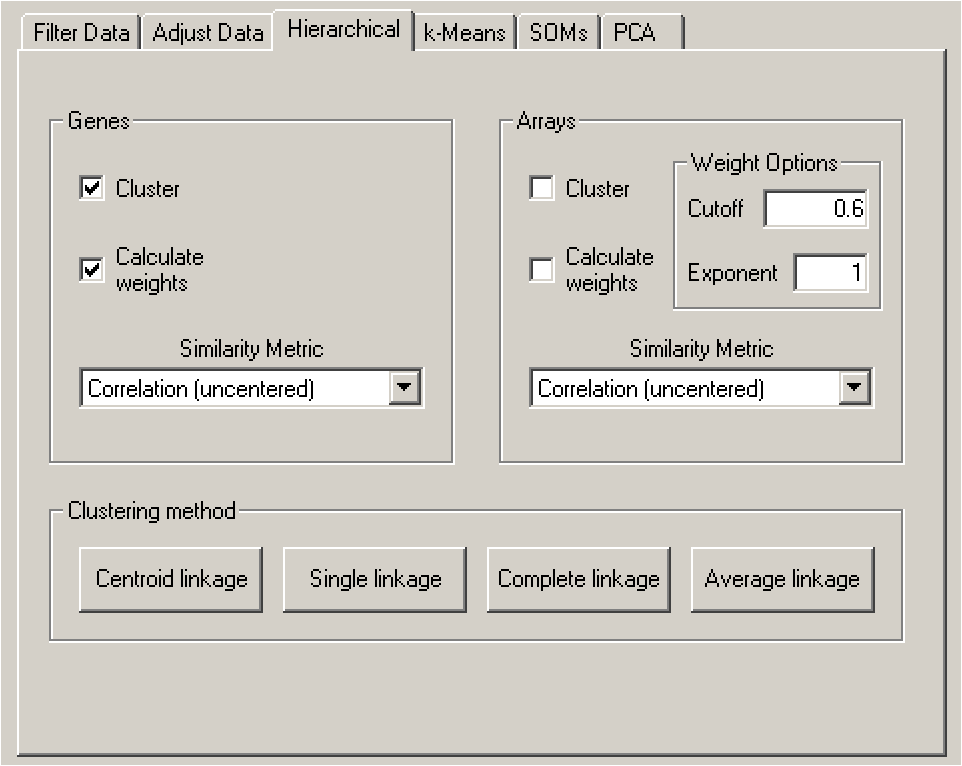

This will expose a Weight Options dialog box in the Arrays panel (I realize this placement is a bit counterintuitive, but it makes sense as you will see below). The idea behind the Calculate Weights option is to weight each row (the same idea applies to columns as well) based on the local density of row vectors in its vicinity, with a high density vicinity resulting in a low weight and a low density vicinity resulting in a higher weight. This is implemented by assigning a local density score $L(i)$ to each row i.

$$L(i) = \sum_{j \text{ with } d(i,j)<k} \left(\frac{k - d(i,j)}{k} \right)^n ,$$

where the cutoff k and the exponent n are user supplied parameters. The weight for each row is $1/L$. Note that $L(i)$ is always at least 1, since $d(i,i) = 0$. Each other row that is within the distance k of row i increases $L(i)$ and decreases the weight. The larger $d(i,j)$, the less $L(i)$ is increased. Values of n greater than 1 mean that the contribution to $L(i)$ drops off rapidly as $d(i,j)$ increases.

4.1.6 Ordering of Output File

The result of a clustering run is a tree or pair of trees (one for genes one for arrays). However, to visualize the results in TreeView, it is necessary to use this tree to reorder the rows and/or columns in the initial datatable. Note that if you simply draw all of the node

in the tree in the following manner, a natural ordering of items emerges:

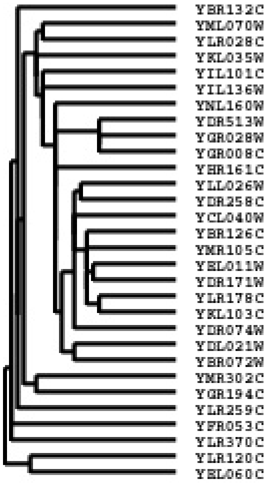

Thus, any tree can be used to generate an ordering. However, the ordering for any given tree is not unique. There is a family of 2^{N-1} ordering consistent with any tree of N items; you can flip any node on the tree (exchange the bottom and top branches) and you will get a new ordering that is equally consistent with the tree. By default, when Cluster joins two items, it randomly places one item on the top branch and the other on the bottom branch. It is possible to guide this process to generate the best ordering consistent with a given tree. This is done by using the '`GORDER`' (gene order) and '`EORDER`' (experiment order) parameters in the input file, or by running a self-organizing map (see section below) prior to clustering. By default, Cluster sets the order parameter for each row/column to 1. When a new node is created, Cluster compares the order parameters of the two joined items, and places the item with the smaller order value on the top branch. The order parameter for a node is the average of the order parameters of its members. Thus, if you want the gene order produced by Cluster to be as close as possible (without violating the structure of the tree) to the order in your input file, you use the '`GORDER`' column, and assign a value that increases for each row. Note that order parameters do not have to be unique.

4.1.7 Output Files

Cluster writes up to three output files for each hierarchical clustering run. The root filename of each file is whatever text you enter into the Job Name dialog box. When you load a file, Job Name is set to the root filename of the input file. The three output files are '*JobName*`.cdt`', '*JobName*`.gtr`', '*JobName*`.atr`' The '`.cdt`' (for clustered data table) file contains the original data with the rows and columns reordered based on the clustering result. It is the same format as the input files, except that an additional column and/or row is added if clustering is performed on genes and/or arrays. This additional column/row contains a unique identifier for each row/column that is linked to the description of the tree structure in the '`.gtr`' and '`.atr`' files. The '`.gtr`' (gene tree) and '`.atr`' (array tree) files are tab-delimited text files that report on the history of node joining in the gene or array clustering (note that these files are produced only when clustering is performed on the corresponding axis). When clustering begins each item to be clustered is assigned a unique identifier (e.g. '`GENE1X`' or '`ARRY42X`' — the '`X`' is a relic from the days when this was written in Perl and substring searches were used). These identifiers are added to the .cdt

file. As each node is generated, it receives a unique identifier as well, starting is '`NODE1X`', '`NODE2X`', etc. Each joining event is stored in the '`.gtr`' or '`.atr`' file as a row with the node identifier, the identifiers of the two joined elements, and the similarity score for the two joined elements. These files look like:

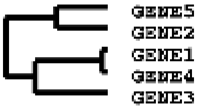

NODE1X	GENE1X	GENE4X	0.98
NODE2X	GENE5X	GENE2X	0.80
NODE3X	NODE1X	GENE3X	0.72
NODE4X	NODE2X	NODE3X	0.60

The '`.gtr`' and/or '`.atr`' files are automatically read in TreeView when you open the corresponding '`.cdt`' file.

4.2 The k-means Clustering Algorithm

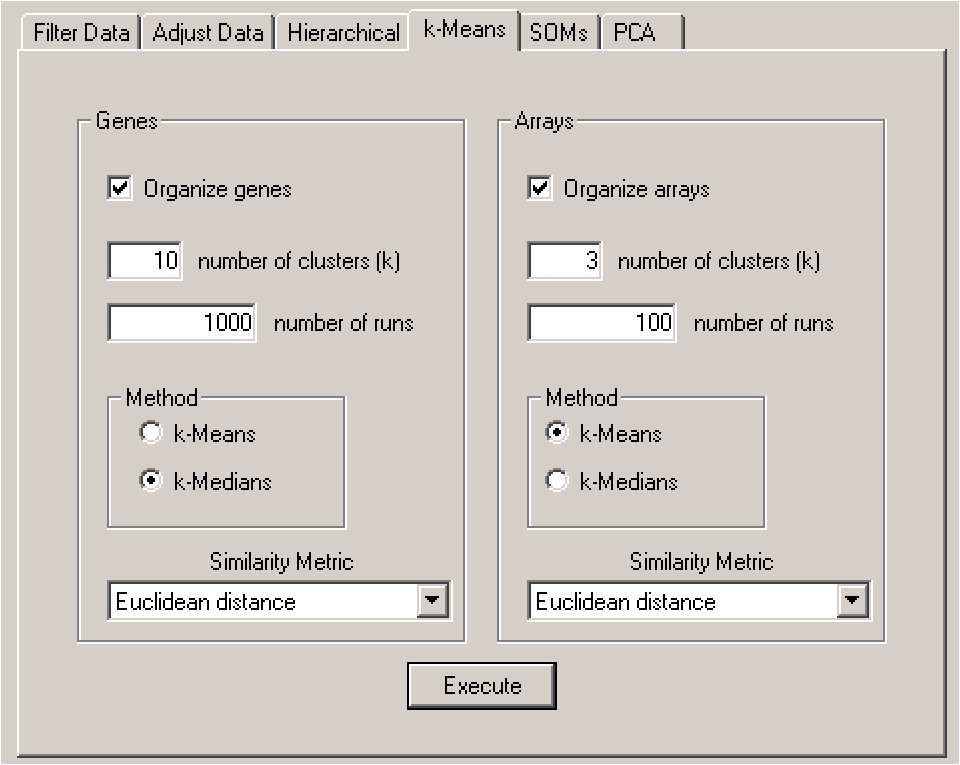

The k-means clustering algorithm is a simple, but popular, form of cluster analysis. The basic idea is that you start with a collection of items (e.g. genes) and some chosen number of clusters (k) you want to find. The items are initially randomly assigned to a cluster. The k-means clustering proceeds by repeated application of a two-step process where

1. the mean vector for all items in each cluster is computed;
2. items are reassigned to the cluster whose center is closest to the item.

Since the initial cluster assignment is random, different runs of the k-means clustering algorithm may not give the same final clustering solution. To deal with this, the k-means clustering algorithms is repeated many times, each time starting from a different initial clustering. The sum of distances within the clusters is used to compare different clustering solutions. The clustering solution with the smallest sum of within-cluster distances is saved.

The number of runs that should be done depends on how difficult it is to find the optimal solution, which in turn depends on the number of genes involved. Cluster therefore shows in the status bar how many times the optimal solution has been found. If this number is one, there may be a clustering solution with an even lower sum of within-cluster distances. The k-means clustering algorithm should then be repeated with more trials. If the optimal solution is found many times, the solution that has been found is likely to have the lowest possible within-cluster sum of distances. We can then assume that the k-means clustering procedure has then found the overall optimal clustering solution.

It should be noted that generally, the k-means clustering algorithm finds a clustering solution with a smaller within-cluster sum of distances than the hierarchical clustering techniques.

The parameters that control k-means clustering are

- the number of clusters (k);
- the number of trials.

The output is simply an assignment of items to a cluster. The implementation here simply rearranges the rows and/or columns based on which cluster they were assigned to. The output data file is '`*JobName*_K_GKg_AKa.cdt`', where '`_GKg`' is included if genes were organized, and '`_AKa`' is included if arrays were organized. Here, '`Kg`' and '`Ka`' represent the number of clusters for gene clustering and array clustering, respectively. This file contains the gene expression data, organized by cluster by rearranging the rows and columns. In addition, the files '`*JobName*_K_GKg.kgg`' and '`*JobName*_K_AKa.kag`' are created, containing a list of genes/arrays and the cluster they were assigned to.

Whereas k-means clustering as implemented in Cluster 3.0 allows any of the eight distance measures to be used, we recommend using the Euclidean distance or city-block distance instead of the distance measures based on the Pearson correlation, for the same reason as in case of pairwise centroid-linkage hierarchical clustering. The distance measures based on the Pearson correlation effectively normalize the data vectors when calculating the distance, whereas no normalization is used when calculating the cluster centroid. To use k-means clustering with a distance measure based on the Pearson correlation, it is better to first normalize the data appropriately (using the `"Adjust Data"` tab) before running the k-means algorithm.

Cluster also implements a slight variation on k-means clustering, known as k-medians clustering, in which the median instead of the mean of items in a node are used. In a theoretical sense, it is best to use k-means with the Euclidean distance, and k-medians with the city-block distance.

4.3 Self-Organizing Maps

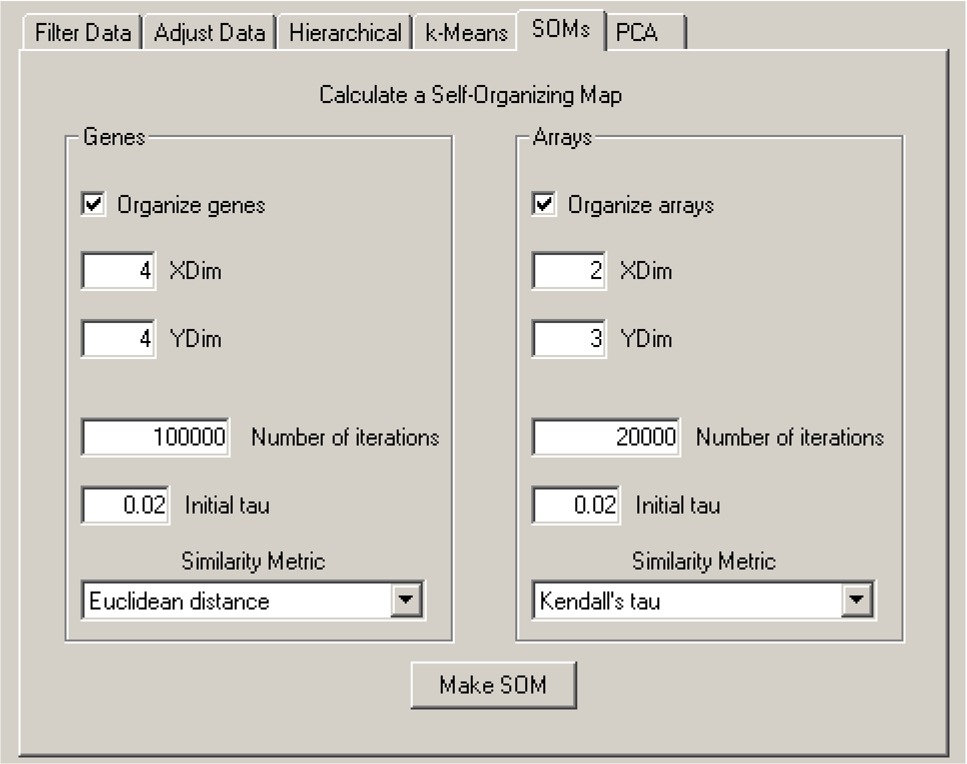

Self-Organizing Maps (SOMs) is a method of cluster analysis that are somewhat related to k-means clustering. SOMs were invented in by Teuvo Kohonen in the early 1980s, and have recently been used in genomic analysis (see Chu 1998, Tamayo 1999 and Golub 1999 in references). The Tamayo paper contains a simple explanation of the methods. A more detailed description is available in the book by Kohonen, Self-Organizing Maps, 1997.

The current implementation varies slightly from that of Tamayo et al., in that it restricts the analysis one-dimensional SOMs along each axis, as opposed to a two-dimensional network. The one-dimensional SOM is used to reorder the elements on whichever axes are selected. The result is similar to the result of k-means clustering, except that, unlike in k-means clustering, the nodes in a SOM are ordered. This tends to result in a relatively smooth transition between groups.

The options for SOMs are

- whether or not you will organize each axis;
- the number of nodes for each axis (the default is $n^{1/4}$, where n is the number of items; the total number of clusters is then equal to the square root of the number of items);
- the number of iterations to be run.

The output file is of the form '*JobName*`_SOM_GXg-Yg_AXa-Ya.txt`', where '`GXg-Yg`' is included if genes were organized, and '`AXg-Yg`' is included if arrays were organized. '`X`' and '`Y`' represent the dimensions of the corresponding SOM. Up to two additional files ('`.gnf`' and '`.anf`') are written containing the vectors for the SOM nodes.

In previous versions of Cluster, only one-dimensional SOMs were supported. The current version of the Cluster introduces two-dimensional SOMs.

SOMs and hierarchical clustering: Our original use of SOMs (see Chu et al., 1998) was motivated by the desire to take advantage of the properties of both SOMs and hierarchical clustering. This was accomplished by first computing a one dimensional SOM, and using the ordering from the SOM to guide the flipping of nodes in the hierarchical tree. In Cluster, after a SOM is run on a dataset, the GORDER and/or EORDER fields are set to the ordering from the SOM so that, for subsequent hierarchical clustering runs, the output ordering will come as close as possible to the ordering in the SOM without violating the structure of the tree.

4.4 Principal Component Analysis

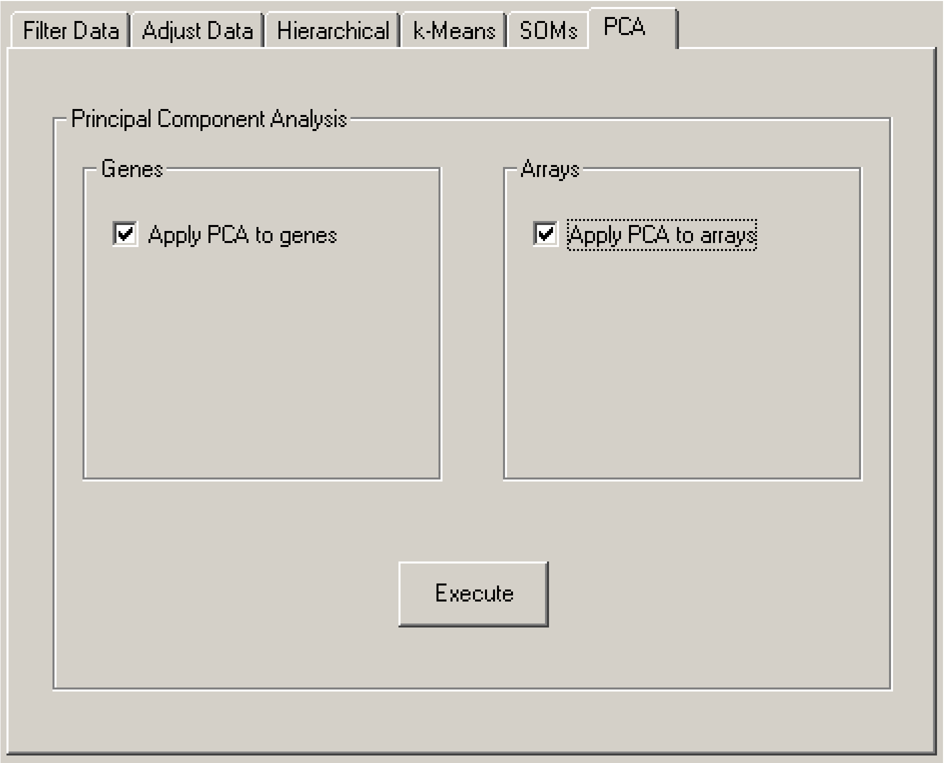

Principal Component Analysis (PCA) is a widely used technique for analyzing multivariate data. A practical example of applying Principal Component Analysis to gene expression data is presented by Yeung and Ruzzo (2001).

In essence, PCA is a coordinate transformation in which each row in the data matrix is written as a linear sum over basis vectors called principal components, which are ordered and chosen such that each maximally explains the remaining variance in the data vectors. For example, an $n \times 3$ data matrix can be represented as an ellipsoidal cloud of n points in three dimensional space. The first principal component is the longest axis of the ellipsoid, the second principal component the second longest axis of the ellipsoid, and the third principal component is the shortest axis. Each row in the data matrix can be reconstructed

as a suitable linear combination of the principal components. However, in order to reduce the dimensionality of the data, usually only the most important principal components are retained. The remaining variance present in the data is then regarded as unexplained variance.

The principal components can be found by calculating the eigenvectors of the covariance matrix of the data. The corresponding eigenvalues determine how much of the variance present in the data is explained by each principal component.

Before applying PCA, typically the mean is subtracted from each column in the data matrix. In the example above, this effectively centers the ellipsoidal cloud around its centroid in 3D space, with the principal components describing the variation of poins in the ellipsoidal cloud with respect to their centroid.

In Cluster, you can apply PCA to the rows (genes) of the data matrix, or to the columns (microarrays) of the data matrix. In each case, the output consists of two files. When applying PCA to genes, the names of the output files are '*JobName*`_pca_gene.pc.txt`' and '*JobName*`_pca_gene.coords.txt`', where the former contains contains the principal components, and the latter contains the coordinates of each row in the data matrix with respect to the principal components. When applying PCA to the columns in the data matrix, the respective file names are '*JobName*`_pca_array.pc.txt`' and '*JobName*`_pca_array.coords.txt`'. The original data matrix can be recovered from the principal components and the coordinates.

As an example, consider this input file:

```
UNIQID   EXP1   EXP2   EXP3
GENE1    3      4      -2
GENE2    4      1      -3
GENE3    1      -8     7
GENE4    -6     6      4
GENE5    0      -3     8
```

Applying PCA to the rows (genes) of the data in this input file generates a coordinate file containing

```
UNIQID   NAME    GWEIGHT    13.513398  10.162987   2.025283
GENE1    GENE1   1.000000   6.280326   -2.404095   -0.760157
GENE2    GENE2   1.000000   4.720801   -4.995230    0.601424
GENE3    GENE3   1.000000   -8.755665  -2.117608    0.924161
GENE4    GENE4   1.000000   3.443490    8.133673    0.621082
GENE5    GENE5   1.000000   -5.688953   1.383261   -1.386509
```

where the first line shows the eigenvalues of the principal components, and a prinpical component file containing

```
EIGVALUE   EXP1        EXP2        EXP3
MEAN        0.400000   0.000000     2.800000
13.513398   0.045493   0.753594    -0.655764
10.162987  -0.756275   0.454867     0.470260
2.025283   -0.652670   -0.474545   -0.590617
```

with the eigenvalues of the principal components shown in the first column. From this principal component decomposition, we can regenerate the original data matrix as follows:

$$\begin{pmatrix} 6.280326 & -2.404095 & -0.760157 \\ 4.720801 & -4.995230 & 0.601424 \\ -8.755665 & -2.117608 & 0.924161 \\ 3.443490 & 8.133673 & 0.621082 \\ -5.688953 & 1.383261 & -1.386509 \end{pmatrix} \cdot \begin{pmatrix} 0.045493 & 0.753594 & -0.655764 \\ -0.756275 & 0.454867 & 0.470260 \\ -0.652670 & -0.474545 & -0.590617 \end{pmatrix}$$

$$+ \begin{pmatrix} 0.400000 & 0.000000 & 2.800000 \\ 0.400000 & 0.000000 & 2.800000 \\ 0.400000 & 0.000000 & 2.800000 \\ 0.400000 & 0.000000 & 2.800000 \\ 0.400000 & 0.000000 & 2.800000 \end{pmatrix} = \begin{pmatrix} 3 & 4 & -2 \\ 4 & 1 & -3 \\ 1 & -8 & 7 \\ -6 & 6 & 4 \\ 0 & -3 & 8 \end{pmatrix}$$

Note that the coordinate file '`JobName_pca_gene.coords.txt`' is a valid input file to Cluster 3.0. Hence, it can be loaded into Cluster 3.0 for further analysis, possibly after removing columns with low eigenvalues.

5 Running Cluster 3.0 as a command line program

Cluster 3.0 can also be run as a command line program. This may be useful if you want to run Cluster 3.0 on a remote server, and also allows automatic processing a large number of data files by running a batch script. Note, however, that the Python and Perl interfaces to the C Clustering Library may be better suited for this task, as they are more powerful than the command line program (see the manual for the C Clustering Library at `http://bonsai.ims.u-tokyo.ac.jp/~mdehoon/software/cluster/cluster.pdf`).

The GUI version of Cluster 3.0 can be used as a command line program by applying the appropriate command line parameters. You can also compile Cluster 3.0 without GUI support (if you will be using it from the command line only) by downloading the source code from `http://bonsai.ims.u-tokyo.ac.jp/~mdehoon/software/cluster`, and running

```
configure --without-x
make
make install
```

The executable is called `cluster`. To run this program, execute

```
cluster [options]
```

in which the options consist of the following command line parameters:

`-f` *`filename`*
: File loading

`-l`
: Specifies to log-transform the data before clustering (default is no log-transform)

`-cg a|m`
: Specifies whether to center each row (gene) in the data set:
`a`: Subtract the mean of each row
`m`: Subtract the median of each row
(default is no centering)

`-ng`
: Specifies to normalize each row (gene) in the data set (default is no normalization)

`-ca a|m`
: Specifies whether to center each column (microarray) in the data set:
`a`: Subtract the mean of each column
`m`: Subtract the median of each column
(default is no centering)

`-na`
: Specifies to normalize each column (microarray) in the data set (default is no normalization)

`-u` *`jobname`*
: Allows you to specify a different name for the output files (default is derived from the input file name)

`-g [0..9]`
: Specifies the distance measure for gene clustering. 0 means no gene clustering; for the values 1 through 9, see below (default: 0)

`-e [0..9]`
: Specifies the distance measure for microarray clustering. 0 means no microarray clustering; for the values 1 through 9, see below (default: 0)

`-m [msca]` Specifies which hierarchical clustering method to use:
`m`: Pairwise complete- (maximum-) linkage (default)
`s`: Pairwise single-linkage
`c`: Pairwise centroid-linkage
`a`: Pairwise average-linkage

`-k` *number*
Specifies whether to run *k*-means clustering instead of hierarchical clustering, and the number of clusters *k* to use (default: 0, no *k*-means clustering)

`-pg` Specifies to apply Principal Component Analysis to genes instead of clustering

`-pa` Specifies to apply Principal Component Analysis to arrays instead of clustering

`-s` Specifies to calculate an SOM instead of hierarchical clustering

`-x` *number*
Specifies the horizontal dimension of the SOM grid (default: 2)

`-y` *number*
Specifies the vertical dimension of the SOM grid (default: 1)

`-v, --version`
Display version information

`-h, --help`
Display help information

For the command line options '`-g`', '`-e`', the following integers can be used to specify the distance measure:

`0` No clustering

`1` Uncentered correlation

`2` Pearson correlation

`3` Uncentered correlation, absolute value

`4` Pearson correlation, absolute value

`5` Spearman's rank correlation

`6` Kendall's tau

`7` Euclidean distance

`8` City-block distance

By default, no clustering is done, allowing you to use `cluster` for normalizing a data set only.

6 TreeView

TreeView is a program that allows interactive graphical analysis of the results from Cluster. TreeView reads in matching '`*.cdt`' and '`*.gtr`', '`*.atr`', '`*.kgg`', or '`*.kag`' files produced by Cluster. We recommend using the Java program Java TreeView, which is based on the original TreeView. Java TreeView was written by Alok Saldanha at Stanford University; it can be downloaded from `http://jtreeview.sourceforge.net/`. Java TreeView runs on Windows, Macintosh, Linux, and Unix computers, and can show both hierarchical and k-means results.

7 Code Development Information

In previous versions of Cluster, the proprietary Numerical Recipes routines were heavily used. We have replaced these routines by the C clustering library, which was released under the Python License. Accordingly, the complete source code of Cluster is now open. It can be downloaded from `http://bonsai.ims.u-tokyo.ac.jp/~mdehoon/software/cluster`. We used the GNU C compiler in order to enable anybody to compile the code. No commercial compiler is required. The GNU C compiler is available at `http://www.gnu.org`. There you can also find texinfo, which was used to generate the printed and the HTML documentation. To convert the picture files to EPS files for the printed documentation, we used `pngtopnm` and `pnmtops` of Netpbm, which can be found at `http://netpbm.sourceforge.net`. The HTML Help file was generated using the HTML Help Workshop, which is freely available at the Microsoft site (`http://msdn.microsoft.com`). The Windows Installer was created with the Inno Setup Compiler, which is available at `http://www.innosetup.com`.

For Mac OS X, we used the Project Builder and the Interface Builder, which are part of the Mac OS X Development Tools. The prebuilt package was created with PackageMaker, which is also part of Mac OS X. The project files needed to recompile Cluster 3.0 are included in the source code. From the command prompt, Cluster 3.0 can be recompiled by running `make` from the `mac` subdirectory; this produces a universal binary for PowerPC and Intel processors.

For Cluster 3.0 on Linux/Unix, we used the Motif libraries that are installed on most Linux/Unix computers. The include files are typically located in `/usr/X11R6/include/Xm`. You will need a version of Motif that is compliant with Motif 2.1, such as Open Motif (`http://www.opengroup.org`), which is available at `http://www.motifzone.net`.

To improve the portability of the code, we made use of GNU's automake and autoconf. The corresponding `Makefile.am` and `configure.ac` files are included in the source code distribution.

8 Bibliography

Brown, P. O., and Botstein, D. (1999). Exploring the new world of the genome with DNA microarrays. *Nat Genet* **21**, 33–37.

Chu, S., DeRisi, J., Eisen, M., Mulholland, J., Botstein, D., Brown, P. O., and Herskowitz, I. (1998). The transcriptional program of sporulation in budding yeast [published erratum appears in *Science* 1998 Nov 20; **282** (5393):1421]. *Science* **282**, 699–705.

Conover, W. J. (1980). *Practical nonparametric statistics* (New York: Wiley).

De Hoon, M., Imoto, S., and Miyano, S. (2002). Statistical analysis of a small set of time-ordered gene expression data using linear splines. *Bioinformatics* **18**, 1477–1485.

De Hoon, M. J. L., Imoto, S., Nolan, J., and Miyano, S. (2004). Open source clustering software. *Bioinformatics*, **20** (9), 1453–1454.

Eisen, M. B., Spellman, P. T., Brown, P. O., and Botstein, D. (1998). Cluster analysis and display of genome-wide expression patterns. *Proc Natl Acad Sci USA* **95**, 14863–14868.

Hartigan, J. A. (1975). *Clustering algorithms* (New York: Wiley).

Jain, A. K., and Dubes, R. C. (1988). *Algorithms for clustering data* (Englewood Cliffs, N.J.: Prentice Hall).

Jardine, N., and Sibson, R. (1971). *Mathematical taxonomy* (London, New York: Wiley).

Kohonen, T. (1997). *Self-organizing maps*, 2nd Edition (Berlin; New York: Springer).

Sibson, R. (1973). SLINK: An optimally efficient algorithm for the single-link cluster method. *The Computer Journal*, **16** (1), 30–34.

Sneath, P. H. A., and Sokal, R. R. (1973). *Numerical taxonomy; the principles and practice of numerical classification* (San Francisco: W. H. Freeman).

Snedecor, G. W. and Cochran, W. G. (1989). *Statistical methods* (Ames: Iowa State University Press).

Sokal, R. R., and Sneath, P. H. A. (1963). *Principles of numerical taxonomy* (San Francisco: W. H. Freeman).

Tamayo, P., Slonim, D., Mesirov, J., Zhu, Q., Kitareewan, S., Dmitrovsky, E., Lander, E., and Golub, T. (1999). Interpreting patterns of gene expression with self-organizing maps: Methods and application to hematopoietic differentiation. *Proc. Natl. Acad. Sci. USA*, **96**, 2907–2912.

Tryon, R. C., and Bailey, D. E. (1970). *Cluster analysis* (New York: McGraw-Hill).

Tukey, J. W. (1977). *Exploratory data analysis* (Reading, Mass.: Addison-Wesley Pub. Co.).

Weinstein, J. N., Myers, T. G., OConnor, P. M., Friend, S. H., Fornace, A. J., Jr., Kohn, K. W., Fojo, T., Bates, S. E., Rubinstein, L. V., Anderson, N. L., Buolamwini, J. K., van Osdol, W. W., Monks, A. P., Scudiero, D. A., Sausville, E. A., Zaharevitz, D. W., Bunow, B., Viswanadhan, V. N., Johnson, G. S., Wittes, R. E., and Paull, K. D. (1997). An information-intensive approach to the molecular pharmacology of cancer. *Science* **275**, 343–349.

Wen, X., Fuhrman, S., Michaels, G. S., Carr, D. B., Smith, S., Barker, J. L., and Somogyi, R. (1998). Large-scale temporal gene expression mapping of central nervous system development. *Proc Natl Acad Sci USA* **95**, 334–339.

Yeung, K. Y., and Ruzzo, W. L. (2001). Principal Component Analysis for clustering gene expression data. *Bioinformatics* **17**, 763–774.
