# Supplementary material for: A genome‐scale screen reveals context‐dependent ovarian cancer sensitivity to miRNA overexpression
Source: Mol Syst Biol. 2015 Dec 11;11(12):842. doi: 10.15252/msb.20156308 (PMC4704493; doi:10.15252/msb.20156308)
Supplement: Supplementary file 12 — Dataset EV8 [file MSB-11-842-s016.zip › Dataset_EV8/Cluster.app/Contents/Resources/html/Bibliography.html]

Bibliography - Cluster 3.0 for Windows, Mac OS X, Linux, Unix


Previous: Development,
Up: Top


---

## 8 Bibliography

Brown, P. O., and Botstein, D. (1999). Exploring the new world of the genome with DNA microarrays. *Nat Genet* **21**, 33–37.

Chu, S., DeRisi, J., Eisen, M., Mulholland, J., Botstein, D., Brown, P. O., and Herskowitz, I. (1998).
The transcriptional program of sporulation in budding yeast
[published erratum appears in *Science* 1998 Nov 20; **282** (5393):1421]. *Science* **282**, 699–705.

Conover, W. J. (1980). *Practical nonparametric statistics* (New York: Wiley).

De Hoon, M., Imoto, S., and Miyano, S. (2002). Statistical analysis of a small set of time-ordered gene expression data using linear splines. *Bioinformatics* **18**, 1477–1485.

De Hoon, M. J. L., Imoto, S., Nolan, J., and Miyano, S. (2004). Open source clustering software. *Bioinformatics*, **20** (9), 1453–1454.

Eisen, M. B., Spellman, P. T., Brown, P. O., and Botstein, D. (1998). Cluster analysis and display of genome-wide expression patterns. *Proc Natl Acad Sci USA* **95**, 14863–14868.

Hartigan, J. A. (1975). *Clustering algorithms* (New York: Wiley).

Jain, A. K., and Dubes, R. C. (1988). *Algorithms for clustering data* (Englewood Cliffs, N.J.: Prentice Hall).

Jardine, N., and Sibson, R. (1971). *Mathematical taxonomy* (London, New York: Wiley).

Kohonen, T. (1997). *Self-organizing maps*, 2nd Edition (Berlin; New York: Springer).

Sibson, R. (1973). SLINK: An optimally efficient algorithm for the single-link cluster method. *The Computer Journal*, **16** (1), 30–34.

Sneath, P. H. A., and Sokal, R. R. (1973). *Numerical taxonomy; the principles and practice of numerical classification* (San Francisco: W. H. Freeman).

Snedecor, G. W. and Cochran, W. G. (1989). *Statistical methods* (Ames: Iowa State University Press).

Sokal, R. R., and Sneath, P. H. A. (1963). *Principles of numerical taxonomy* (San Francisco: W. H. Freeman).

Tamayo, P., Slonim, D., Mesirov, J., Zhu, Q., Kitareewan, S., Dmitrovsky, E., Lander, E., and Golub, T. (1999). Interpreting patterns of gene expression with self-organizing maps: Methods and application to hematopoietic differentiation. *Proc. Natl. Acad. Sci. USA*, **96**, 2907–2912.

Tryon, R. C., and Bailey, D. E. (1970). *Cluster analysis* (New York: McGraw-Hill).

Tukey, J. W. (1977). *Exploratory data analysis* (Reading, Mass.: Addison-Wesley Pub.
Co.).

Weinstein, J. N., Myers, T. G., OConnor, P. M., Friend, S. H., Fornace, A. J., Jr., Kohn, K. W., Fojo, T., Bates, S. E., Rubinstein, L. V., Anderson, N. L., Buolamwini, J. K., van Osdol, W. W., Monks, A. P., Scudiero, D. A., Sausville, E. A., Zaharevitz, D. W., Bunow, B., Viswanadhan, V. N., Johnson, G. S., Wittes, R. E., and Paull, K. D. (1997).
An information-intensive approach to the molecular pharmacology of cancer.
*Science* **275**, 343–349.

Wen, X., Fuhrman, S., Michaels, G. S., Carr, D. B., Smith, S., Barker, J. L., and Somogyi, R. (1998).
Large-scale temporal gene expression mapping of central nervous system development.
*Proc Natl Acad Sci USA* **95**, 334–339.

Yeung, K. Y., and Ruzzo, W. L. (2001). Principal Component Analysis for clustering gene expression data.
*Bioinformatics* **17**, 763–774.
