# Supplementary material for: A genome‐scale screen reveals context‐dependent ovarian cancer sensitivity to miRNA overexpression
Source: Mol Syst Biol. 2015 Dec 11;11(12):842. doi: 10.15252/msb.20156308 (PMC4704493; doi:10.15252/msb.20156308)
Supplement: Supplementary file 12 — Dataset EV8 [file MSB-11-842-s016.zip › Dataset_EV8/Cluster.app/Contents/Resources/html/Cluster.html]

Cluster - Cluster 3.0 for Windows, Mac OS X, Linux, Unix


Next: Command,
Previous: Distance,
Up: Top


---

## 4 Clustering techniques

The Cluster program provides several clustering algorithms. Hierarchical clustering methods organizes genes in a tree structure, based on their similarity. Four variants of hierarchical clustering are available in Cluster. In
*k*-means clustering, genes are organized into
*k* clusters, where the number of clusters
*k* needs to be chosen in advance.
Self-Organizing Maps create clusters of genes on a two-dimensional rectangular grid, where neighboring clusters are similar. For each of these methods, one of the eight different distance meaures can be used. Finally, in Principal Component Analysis, clusters are organized based on the principal component axes of the distance matrix.

- Hierarchical: Pairwise single-, maximum-, average-, and centroid-linkage hierarchical clustering- KMeans: *k*-means and *k*-medoids clusteringclustering- SOM: Self-Organizing Maps- PCA: Principal Component Analysis
