# Supplementary material for: A genome‐scale screen reveals context‐dependent ovarian cancer sensitivity to miRNA overexpression
Source: Mol Syst Biol. 2015 Dec 11;11(12):842. doi: 10.15252/msb.20156308 (PMC4704493; doi:10.15252/msb.20156308)
Supplement: Supplementary file 12 — Dataset EV8 [file MSB-11-842-s016.zip › Dataset_EV8/Cluster.app/Contents/Resources/html/Command.html]

Command - Cluster 3.0 for Windows, Mac OS X, Linux, Unix


Next: TreeView,
Previous: Cluster,
Up: Top


---

## 5 Running Cluster 3.0 as a command line program

Cluster 3.0 can also be run as a command line program. This may be useful if you want to run Cluster 3.0 on a remote server, and also allows automatic processing a large number of data files by running a batch script. Note, however, that the Python and Perl interfaces to the C Clustering Library may be better suited for this task, as they are more powerful than the command line program (see the manual for the C Clustering Library at http://bonsai.ims.u-tokyo.ac.jp/~mdehoon/software/cluster/cluster.pdf).

The GUI version of Cluster 3.0 can be used as a command line program by applying the appropriate command line parameters. You can also compile Cluster 3.0 without GUI support (if you will be using it from the command line only) by downloading the source code from http://bonsai.ims.u-tokyo.ac.jp/~mdehoon/software/cluster, and running   
`configure --without-x`   
`make`   
`make install`   
The executable is called `cluster`. To run this program, execute   
`cluster [options]`   
in which the options consist of the following command line parameters:

`-f` filename: File loading `-l`: Specifies to log-transform the data before clustering (default is no log-transform) `-cg a|m`: Specifies whether to center each row (gene) in the data set: `a`: Subtract the mean of each row `m`: Subtract the median of each row (default is no centering) `-ng`: Specifies to normalize each row (gene) in the data set (default is no normalization) `-ca a|m`: Specifies whether to center each column (microarray) in the data set: `a`: Subtract the mean of each column `m`: Subtract the median of each column (default is no centering) `-na`: Specifies to normalize each column (microarray) in the data set (default is no normalization) `-u` jobname: Allows you to specify a different name for the output files (default is derived from the input file name) `-g [0..9]`: Specifies the distance measure for gene clustering. 0 means no gene clustering; for the values 1 through 9, see below (default: 0) `-e [0..9]`: Specifies the distance measure for microarray clustering. 0 means no microarray clustering; for the values 1 through 9, see below (default: 0) `-m [msca]`: Specifies which hierarchical clustering method to use: `m`: Pairwise complete- (maximum-) linkage (default) `s`: Pairwise single-linkage `c`: Pairwise centroid-linkage `a`: Pairwise average-linkage `-k` number: Specifies whether to run *k*-meansclustering instead of hierarchical clustering, and the number of clusters *k* to use (default: 0, no *k*-means clustering) `-pg`: Specifies to apply Principal Component Analysis to genes instead of clustering `-pa`: Specifies to apply Principal Component Analysis to arrays instead of clustering `-s`: Specifies to calculate an SOM instead of hierarchical clustering `-x` number: Specifies the horizontal dimension of the SOM grid (default: 2) `-y` number: Specifies the vertical dimension of the SOM grid (default: 1) `-v, --version`: Display version information `-h, --help`: Display help information

For the command line options -g, -e, the following integers can be used to specify the distance measure:

`0`: No clustering `1`: Uncentered correlation `2`: Pearson correlation `3`: Uncentered correlation, absolute value `4`: Pearson correlation, absolute value `5`: Spearman's rank correlation `6`: Kendall's tau `7`: Euclidean distance `8`: City-block distance

By default, no clustering is done, allowing you to use `cluster` for normalizing a data set only.
