# Supplementary material for: A genome‐scale screen reveals context‐dependent ovarian cancer sensitivity to miRNA overexpression
Source: Mol Syst Biol. 2015 Dec 11;11(12):842. doi: 10.15252/msb.20156308 (PMC4704493; doi:10.15252/msb.20156308)
Supplement: Supplementary file 12 — Dataset EV8 [file MSB-11-842-s016.zip › Dataset_EV8/Cluster.app/Contents/Resources/html/Contents.html]

Contents - Cluster 3.0 for Windows, Mac OS X, Linux, Unix


Next: Introduction,
Previous: Top,
Up: Top


---

## Table of Contents

- 1 Introduction- 2 Loading, filtering, and adjusting data
    - 2.1 Loading Data- 2.2 Filtering Data- 2.3 Adjusting Data
          - 2.3.1 Log transformation- 2.3.2 Mean/Median Centering- 2.3.3 Normalization- 3 Distance/Similarity measures
      - 3.1 Distance measures based on the Pearson correlation- 3.2 Non-parametric distance measures- 3.3 Distance measures related to the Euclidean distance
            - 3.3.1 Euclidean distance- 3.3.2 City-block distance- 3.4 Missing values- 3.5 Calculating the distance matrix- 4 Clustering techniques
        - 4.1 Hierarchical Clustering
          - 4.1.1 Centroid Linkage Clustering- 4.1.2 Single Linkage Clustering- 4.1.3 Complete Linkage Clustering- 4.1.4 Average Linkage Clustering- 4.1.5 Weighting- 4.1.6 Ordering of Output File- 4.1.7 Output Files- 4.2 The *k*-means Clustering Algorithm- 4.3 Self-Organizing Maps- 4.4 Principal Component Analysis- 5 Running Cluster 3.0 as a command line program- 6 TreeView- 7 Code Development Information- 8 Bibliography
