# Supplementary material for: A genome‐scale screen reveals context‐dependent ovarian cancer sensitivity to miRNA overexpression
Source: Mol Syst Biol. 2015 Dec 11;11(12):842. doi: 10.15252/msb.20156308 (PMC4704493; doi:10.15252/msb.20156308)
Supplement: Supplementary file 12 — Dataset EV8 [file MSB-11-842-s016.zip › Dataset_EV8/Cluster.app/Contents/Resources/html/Data.html]

Data - Cluster 3.0 for Windows, Mac OS X, Linux, Unix


Next: Distance,
Previous: Introduction,
Up: Top


---

## 2 Loading, filtering, and adjusting data

Data can be loaded into Cluster by choosing Load data file under the File menu.
A number of options are provided for adjusting and
filtering the data you have loaded. These functions are accessed via the Filter Data and
Adjust Data tabs.

### 2.1 Loading Data

The first step in using Cluster is to import data. Currently, Cluster only reads tab-delimited text files in a particular format, described below. Such tab-delimited text files can be created and exported in any standard spreadsheet program, such as Microsoft Excel. An example datafile can be found under the File format help item in the Help menu. This contains all the information you need for making a Cluster input file.

By convention, in Cluster input tables rows represent genes and columns represent samples or observations (e.g. a single microarray hybridization). For a simple timecourse, a minimal Cluster input file would look like this:  
  
Each row (gene) has an identifier (in green) that always goes in the first column. Here we are using yeast open reading frame codes. Each column (sample) has a label (in blue) that is always in the first row; here the labels describe the time at which a sample was taken. The first column of the first row contains a special field (in red) that tells the program what kind of objects are in each row. In this case, YORF stands for yeast open reading frame. This field can be any alpha-numeric value. It is used in TreeView to specify how rows are linked to external websites.

The remaining cells in the table contain data for the appropriate gene and sample. The 5.8 in row 2 column 4 means that the observed data value for gene YAL001C at 2 hours was 5.8. Missing values are acceptable and are designated by empty cells (e.g. YAL005C at 2 hours).

It is possible to have additional information in the input file. A maximal Cluster input file would look like this:  
  
The yellow columns and rows are optional. By default, TreeView uses the ID in column
1 as a label for each gene. The NAME column allows you to specify a label for each gene
that is distinct from the ID in column 1. The other rows and columns will be described
later in this text.

When Cluster 3.0 opens the data file, the number of columns in each row is checked. If a given row contains less or more columns than needed, an error message is displayed.  

### Demo data

A demo datafile, which will be used in all of the examples here, is available
at http://rana.lbl.gov/downloads/data/demo.txt and is mirrored at http://bonsai.ims.u-tokyo.ac.jp/~mdehoon/software/cluster/demo.txt.

The datafile contains yeast gene expression data
described in Eisen *et al.* (1998) [see references at end]. Download this data and load it
into Cluster. Cluster will give you information about the loaded datafile.   

### 2.2 Filtering Data

The Filter Data tab allows you to remove genes that do not have certain desired
properties from your dataset. The currently available properties that can be used to filter
data are

- **% Present >= X**. This removes all genes that have missing values in greater than
  (100-*X*) percent of the columns.- **SD (Gene Vector) >= X**. This removes all genes that have standard deviations of
    observed values less than
    *X*.- **At least X Observations with abs(Val) >= Y**. This removes all genes that do not have at least
      *X* observations with absolute values greater than
      *Y*.- **MaxVal-MinVal >= X**. This removes all genes whose maximum minus minimum values
        are less than
        *X*.

These are fairly self-explanatory. When you press filter, the filters are not immediately
applied to the dataset. You are first told how many genes would have passed the filter. If
you want to accept the filter, you press Accept, otherwise no changes are made.
  

### 2.3 Adjusting Data

From the Adjust Data tab, you can perform a number of operations that alter the
underlying data in the imported table. These operations are

- **Log Transform Data**: replace all data values
  *x* by
  log2 (*x*).- **Center genes [mean or median]**: Subtract the row-wise mean or median from the values in each row of data, so that the mean or median value of each row is 0.- **Center arrays [mean or median]**: Subtract the column-wise mean or median from the values in each column of data, so that the mean or median value of each column is 0.- **Normalize genes**: Multiply all values in each row of data by a scale factor
        *S* so that the sum of the squares of the values in each row is 1.0 (a separate
        *S* is computed for each row).- **Normalize arrays**: Multiply all values in each column of data by a scale factor
          *S* so that the sum of the squares of the values in each column is 1.0 (a separate
          *S* is computed for each column).

These operations are not associative, so the order in which these operations is applied is
very important, and you should consider it carefully before you apply these operations.
The order of operations is (only checked operations are performed):

- Log transform all values.- Center rows by subtracting the mean or median.- Normalize rows.- Center columns by subtracting the mean or median.- Normalize columns.

#### 2.3.1 Log transformation

The results of many DNA microarray experiments are fluorescent
ratios. Ratio measurements are most naturally processed in log space. Consider an
experiment where you are looking at gene expression over time, and the results are
relative expression levels compared to time 0. Assume at timepoint 1, a gene is
unchanged, at timepoint 2 it is up 2-fold and at timepoint three is down 2-fold relative to
time 0. The raw ratio values are 1.0, 2.0 and 0.5. In most applications, you want to think
of 2-fold up and 2-fold down as being the same magnitude of change, but in an opposite
direction. In raw ratio space, however, the difference between timepoint 1 and 2 is +1.0,
while between timepoint 1 and 3 is -0.5. Thus mathematical operations that use the
difference between values would think that the 2-fold up change was twice as significant
as the 2-fold down change. Usually, you do not want this. In log space (we use log base 2
for simplicity) the data points become 0,1.0,-1.0.With these values, 2-fold up and 2-fold
down are symmetric about 0. For most applications, we recommend you work in log
space.

#### 2.3.2 Mean/Median Centering

Consider a now common experimental design where you are
looking at a large number of tumor samples all compared to a common reference sample
made from a collection of cell-lines. For each gene, you have a series of ratio values that
are relative to the expression level of that gene in the reference sample. Since the
reference sample really has nothing to do with your experiment, you want your analysis
to be independent of the amount of a gene present in the reference sample. This is
achieved by adjusting the values of each gene to reflect their variation from some
property of the series of observed values such as the mean or median. This is what mean
and/or median centering of genes does. Centering makes less sense in experiments where
the reference sample is part of the experiment, as it is many timecourses.
Centering the data for columns/arrays can also be used to remove certain types of biases.
The results of many two-color fluorescent hybridization experiments are not corrected for
systematic biases in ratios that are the result of differences in RNA amounts, labeling
efficiency and image acquisition parameters. Such biases have the effect of multiplying
ratios for all genes by a fixed scalar. Mean or median centering the data in log-space has
the effect of correcting this bias, although it should be noted that an assumption is being
made in correcting this bias, which is that the average gene in a given experiment is
expected to have a ratio of 1.0 (or log-ratio of 0).

In general, I recommend the use of median rather than mean centering, as it is more robust against outliers.

#### 2.3.3 Normalization

Normalization sets the magnitude (sum of the squares of the values) of a row/column
vector to 1.0. Most of the distance metrics used by Cluster work with internally
normalized data vectors, but the data are output as they were originally entered. If you
want to output normalized vectors, you should select this option.
A sample series of operations for raw data would be:

- Adjust Cycle 1) log transform- Adjust Cycle 2) median center genes and arrays- repeat (2) five to ten times- Adjust Cycle 3) normalize genes and arrays- repeat (3) five to ten times

This results in a log-transformed, median polished (i.e. all row-wise and column-wise
median values are close to zero) and normal (i.e. all row and column magnitudes are
close to 1.0) dataset.
After performing these operations you should save the dataset.
