# Supplementary material for: A genome‐scale screen reveals context‐dependent ovarian cancer sensitivity to miRNA overexpression
Source: Mol Syst Biol. 2015 Dec 11;11(12):842. doi: 10.15252/msb.20156308 (PMC4704493; doi:10.15252/msb.20156308)
Supplement: Supplementary file 12 — Dataset EV8 [file MSB-11-842-s016.zip › Dataset_EV8/Cluster.app/Contents/Resources/html/Development.html]

Development - Cluster 3.0 for Windows, Mac OS X, Linux, Unix


Next: Bibliography,
Previous: TreeView,
Up: Top


---

## 7 Code Development Information

In previous versions of Cluster, the proprietary Numerical Recipes routines were heavily used. We have replaced these routines by the C clustering library, which was released under the Python License. Accordingly, the complete source code of Cluster is now open.
It can be downloaded from http://bonsai.ims.u-tokyo.ac.jp/~mdehoon/software/cluster. We used the GNU C compiler in order to enable anybody to compile the code. No commercial compiler is required. The GNU C compiler is available at http://www.gnu.org.
There you can also find texinfo, which was used to generate the printed and the HTML documentation. To convert the picture files to EPS files for the printed documentation, we used `pngtopnm` and `pnmtops` of Netpbm, which can be found at http://netpbm.sourceforge.net.
The HTML Help file was generated using the HTML Help Workshop, which is freely available at the Microsoft site.
The Windows Installer was created with the Inno Setup Compiler, which is available at http://www.innosetup.com.

For Mac OS X, we used the Project Builder and the Interface Builder, which are part of the Mac OS X Development Tools. The prebuilt package was created with PackageMaker, which is also part of Mac OS X. The project files needed to recompile Cluster 3.0 are included in the source code. From the command prompt, Cluster 3.0 can be recompiled by running `make` from the `mac` subdirectory; this produces a universal binary for PowerPC and Intel processors.

For Cluster 3.0 on Linux/Unix, we used the Motif libraries that are installed on most Linux/Unix computers. The include files are typically located in `/usr/X11R6/include/Xm`. You will need a version of Motif that is compliant with Motif 2.1, such as Open Motif (http://www.opengroup.org), which is available at http://www.motifzone.net.

To improve the portability of the code, we made use of GNU's automake and autoconf. The corresponding `Makefile.am` and `configure.ac` files are included in the source code distribution.
