# Supplementary material for: A genome‐scale screen reveals context‐dependent ovarian cancer sensitivity to miRNA overexpression
Source: Mol Syst Biol. 2015 Dec 11;11(12):842. doi: 10.15252/msb.20156308 (PMC4704493; doi:10.15252/msb.20156308)
Supplement: Supplementary file 12 — Dataset EV8 [file MSB-11-842-s016.zip › Dataset_EV8/Cluster.app/Contents/Resources/html/Distance.html]

Distance - Cluster 3.0 for Windows, Mac OS X, Linux, Unix


Next: Cluster,
Previous: Data,
Up: Top


---

## 3 Distance/Similarity measures

The first choice that must be made is how similarity (or alternatively, distance) between gene expression data is to be defined. There are many
ways to compute how similar two series of numbers are. Cluster provides eight
options.

### 3.1 Distance measures based on the Pearson correlation

The most commonly used similarity metrics are based on Pearson
correlation.
The Pearson correlation coefficient between any two series of numbers
*x* = {*x*1, *x*2, ..., *xn*} and
*y* = {*y*1, *y*2, ..., *yn*} is defined as

|  |  |  |  |  |  |  |  |  |  |  |  |  |  |  |  |  |  |  |  |  |  |
| --- | --- | --- | --- | --- | --- | --- | --- | --- | --- | --- | --- | --- | --- | --- | --- | --- | --- | --- | --- | --- | --- |
| *r* = | |  | | --- | | 1 | | |  | | --- | |  | | | *n* | |  | *n*  ∑  *i* = 1 | ( | |  | | --- | | *xi* - *x* | | |  | | --- | |  | | | *σx* | | ) | ( | |  | | --- | | *yi* - *y* | | |  | | --- | |  | | | *σy* | | ) |

where
*x* is the average of values in
*x* and
σ*x* is the standard deviation of these values.

There are many ways of conceptualizing the correlation coefficient. If you were to make
a scatterplot of the values of
*x* against
*y* (pairing
*x*1 with
*y*1, *x*2 with
*y*2, etc), then
*r* reports how well you can fit a line to the values.

The simplest way to think about the correlation coefficient is to plot
*x* and
*y* as curves, with
*r* telling you how similar the shapes of the two curves are.
The Pearson correlation coefficient is always between -1 and 1, with 1 meaning that the two
series are identical, 0 meaning they are completely uncorrelated, and -1 meaning they are
perfect opposites. The correlation coefficient is invariant under linear transformation of
the data. That is, if you multiply all the values in
*y* by 2, or add 7 to all the values in
*y*, the correlation between
*x* and
*y* will be unchanged. Thus, two curves that have identical shape, but different
magnitude, will still have a correlation of 1.

Cluster actually uses four different flavors of the Pearson correlation. The textbook
Pearson correlation coefficient, given by the formula above, is used if you select
Correlation (centered) in the Similarity Metric dialog box.
Correlation (uncentered) uses the following modified equations:  

|  |  |  |  |  |  |  |  |  |  |  |  |  |  |  |  |  |  |  |  |  |  |
| --- | --- | --- | --- | --- | --- | --- | --- | --- | --- | --- | --- | --- | --- | --- | --- | --- | --- | --- | --- | --- | --- |
| *r* = | |  | | --- | | 1 | | |  | | --- | |  | | | *n* | |  | *n*  ∑  *i* = 1 | ( | |  | | --- | | *xi* | | |  | | --- | |  | | | *σx*(0) | | ) | ( | |  | | --- | | *yi* | | |  | | --- | |  | | | *σy*(0) | | ) |

  
in which  

|  |  |  |  |  |  |  |  |  |  |  |  |
| --- | --- | --- | --- | --- | --- | --- | --- | --- | --- | --- | --- |
| *σx*(0) = | √ | ( | |  | | --- | | 1 | | |  | | --- | |  | | | *n* | |  | *n*  ∑  *i* = 1 | *xi*2 | ) |

|  |  |  |  |  |  |  |  |  |  |  |  |
| --- | --- | --- | --- | --- | --- | --- | --- | --- | --- | --- | --- |
| *σy*(0) = | √ | ( | |  | | --- | | 1 | | |  | | --- | |  | | | *n* | |  | *n*  ∑  *i* = 1 | *yi*2 | ) |

  
This is basically the same function, except that it assumes the mean is 0, even when it is not. The difference is that, if you have two vectors
*x* and
*y* with identical shape, but which are offset relative to each other by a fixed value, they will have a standard Pearson correlation (centered correlation) of 1 but will not have an uncentered correlation of 1.
The uncentered correlation is equal to the cosine of the angle of two
*n*-dimensional vectors
*x* and
*y*, each representing a vector in
*n*-dimensional space that passes through the origin.
Cluster provides two similarity metrics that are the absolute value of these two correlation functions, which consider two items to be similar if they have opposite expression patterns; the standard correlation coefficients consider opposite genes to be very distant.

### 3.2 Non-parametric distance measures

The Spearman rank correlation and Kendall's
τ are two additional metrics, which are non-parametric versions of the Pearson correlation coefficient. These methods are more robust against outliers.

The Spearman rank correlation calculates the correlation between the ranks of the data values in the two vectors. For example, if we have two data vectors  

*x* = {2.3, 6.7, 4.5, 20.8};  
*y* = {2.1, 5.9, 4.4, 4.2},

then we first replace them by their ranks:  

*x* = {1, 3, 2, 4};  
*y* = {1, 4, 3, 2}.

Now we calculate the correlation coefficient in their usual manner from these data vectors, resulting in  

*r*Spearman = 0.4.

In comparison, the regular Pearson correlation between these data is
*r* = 0.2344. By replacing the data values by their ranks, we reduced the effect of the outlier 20.8 on the value of the correlation coefficient. The Spearman rank correlation can be used as a test statistic for independence between
*x* and *y*. For more information, see Conover (1980).

Kendall's
τ goes a step further by using only the relative ordering of
*x* and *y* to calculate the correlation (Snedecor & Cochran). To calculate Kendall's
τ, consider all pairs of data points
(*xi*, *yi*) and (*xj*, *yj*). We call a pair concordant if

- *xi* < *xj* and *yi* < *yj*; or- *xi* > *xj* and *yi* > *yj*,

and discordant if

- *xi* < *xj* and *yi* > *yj*; or- *xi* > *xj* and *yi* < *yj*.

We can represent this by a table:

|  |  |  |  |  |
| --- | --- | --- | --- | --- |
| - | (2.3, 2.1) | (6.7, 5.9) | (4.5, 4.4) | (20.8, 4.2) |
| (2.3, 2.1) | - | << | << | << |
| (6.7, 5.9) | >> | - | >> | <> |
| (4.5, 4.4) | >> | << | - | <> |
| (20.8, 4.2) | >> | >< | >< | - |

From this table, we find that there are four concordant pairs and two discordant pairs:

*n*c = 4;  
*n*d = 2.

Kendall's
*τ* is calculated as  

|  |  |  |  |  |  |  |  |
| --- | --- | --- | --- | --- | --- | --- | --- |
| *τ* = |  | |  | | --- | | *n*c-*n*d | | |  | | --- | |  | | | *n*(*n*-1)/2 | | , |

which in this case evaluates as 0.33. In the C Clustering Library, the calculation of Kendall's
*τ* is corrected for the possibility that two ranks are equal.
As in case of the Spearman rank correlation, we may use Kendall's
*τ* to test for independence between
*x* and *y*.

### 3.3 Distance measures related to the Euclidean distance

#### 3.3.1 Euclidean distance

A newly added distance function is the Euclidean distance, which is defined as

|  |  |  |  |  |  |  |  |  |  |  |
| --- | --- | --- | --- | --- | --- | --- | --- | --- | --- | --- |
| *d* = | |  | | --- | | 1 | | |  | | --- | |  | | | *n* | |  | *n*  ∑  *i* = 1 | ( | *xi* - *yi* | )2 |

The Euclidean distance takes the difference between two gene expression levels directly. It should therefore only be used for expression data that are suitably normalized, for example by converting the measured gene expression levels to log-ratios. In the sum, we only include terms for which both
*xi* and *yi* are present, and divide by
*n* accordingly.

Unlike the correlation-based distance measures, the Euclidean distance takes the magnitude of changes in the gene expression levels into account. An example of the Euclidean distance applied to
*k*-means clustering can be found in De Hoon, Imoto, and Miyano (2002).

#### 3.3.2 City-block distance

The city-block distance, alternatively known as the Manhattan distance, is related to the Euclidean distance. Whereas the Euclidean distance corresponds to the length of the shortest path between two points, the city-block distance is the sum of distances along each dimension:

|  |  |  |  |  |  |  |  |  |  |  |
| --- | --- | --- | --- | --- | --- | --- | --- | --- | --- | --- |
| *d* = | |  | | --- | | 1 | | |  | | --- | |  | | | *n* | |  | *n*  ∑  *i* = 1 | | | *xi* - *yi* | | |

This is equal to the distance you would have to walk between two points in a city, where you have to walk along city blocks. The city-block distance is a metric, as it satisfies the triangle inequality. Again we only include terms for which both
*xi* and *yi* are present, and divide by
*n* accordingly.

As for the Euclidean distance, the expression data are subtracted directly from each other, and we should therefore make sure that they are properly normalized.

### 3.4 Missing values

When either
*x* or
*y* has missing values, only observations present for both
*x* and
*y* are used in computing similarities.

### 3.5 Calculating the distance matrix

With any specified metric, the first step in the hierarchical clustering routines described below is to compute the
distance (the opposite of similarity; for all correlation metrics distance = 1.0 - correlation)
between all pairs of items to be clustered (e.g. the set of genes in the current dataset).
This can often be time consuming, and, except for pairwise single-linkage clustering,
memory intensive (the maximum amount of memory required is
4 x *N* x *N* bytes, where
*N* is the number of items being clustered). The algorithm for pairwise single-linkage hierarchical clustering is less memory-intensive (linear in
*N*).
