# Supplementary material for: A genome‐scale screen reveals context‐dependent ovarian cancer sensitivity to miRNA overexpression
Source: Mol Syst Biol. 2015 Dec 11;11(12):842. doi: 10.15252/msb.20156308 (PMC4704493; doi:10.15252/msb.20156308)
Supplement: Supplementary file 12 — Dataset EV8 [file MSB-11-842-s016.zip › Dataset_EV8/Cluster.app/Contents/Resources/html/Hierarchical.html]

Hierarchical - Cluster 3.0 for Windows, Mac OS X, Linux, Unix


Next: KMeans,
Up: Cluster


---

### 4.1 Hierarchical Clustering

The Hierarchical Clustering tab allows you to perform hierarchical clustering on your
data. This is a powerful and useful method for analyzing all sorts of large
genomic datasets. Many published applications of this analysis are given in the references
section at the end.

Cluster currently performs four types of binary, agglomerative, hierarchical clustering.
The basic idea is to assemble a set of items (genes or arrays) into a tree, where items are
joined by very short branches if they are very similar to each other, and by increasingly
longer branches as their similarity decreases.

The first step in hierarchical clustering is to calculate the distance matrix between the gene expression data. Once this matrix of distances is computed, the clustering begins.
Agglomerative hierarchical processing consists of repeated cycles where
the two closest remaining items (those with the smallest distance) are joined by a
node/branch of a tree, with the length of the branch set to the distance between the joined
items. The two joined items are removed from list of items being processed and replaced by a
item that represents the new branch. The distances between this new item and all other
remaining items are computed, and the process is repeated until only one item remains.

Note that once clustering commences, we are working with items that are true items (e.g.
a single gene) and items that are pseudo-items that contain a number of true items. There
are a variety of ways to compute distances when we are dealing with pseudo-items, and
Cluster currently provides four choices, which are called centroid linkage, single linkage, complete linkage, and average linkage.
**Note that in older versions of Cluster, centroid linkage was referred to as average linkage.**

#### 4.1.1 Centroid Linkage Clustering

If you click Centroid Linkage Clustering, a vector is assigned to each pseudo-item, and this vector is used to compute the distances between this pseudo-item and all remaining items or pseudo-items using the same similarity metric as was used to calculate the initial similarity matrix. The vector is the average of the vectors of all actual items (e.g. genes) contained within the pseudo-item. Thus, when a new branch of the tree is formed joining together a branch with 5 items and an actual item, the new pseudo-item is assigned a vector that is the average of the 6 vectors it contains, and not the average of the two joined items (note that missing values are not used in the average, and a pseudo-item can have a missing value if all of the items it contains are missing values in the corresponding row/column).

Note that from a theoretical perspective, Centroid Linkage Clustering is peculiar if it is used in combination with one of the distance measures that are based on the Pearson correlation. For these distance measures, the data vectors are implicitly normalized when calculating the distance (for example, by subtracting the mean and dividing by the standard deviation when calculating the Pearson correlation. However, when two genes are joined and their centroid is calculated by averaging their data vectors, no normalization is applied. This may lead to the surprising result that distances may decrease when we go up in the tree representing the hierarchical clustering result. For example, consider this data set:

Exp 1 | Exp 2 | Exp 3 | Exp 4 | || Gene 1 | 0.96 | 0.07 | 0.97 | 0.98 |
| Gene 2 | 0.50 | 0.28 | 0.29 | 0.77 |
| Gene 3 | 0.08 | 0.96 | 0.51 | 0.51 |
| Gene 4 | 0.14 | 0.19 | 0.41 | 0.51 |

Performing pairwise centroid-linkage hierarchical clustering on this data set, using the Pearson distance as the distance measure, produces the clustering result

- Gene 1 joins Gene 2 at distance 0.47- (Gene 1, Gene 2) joins Gene 4 at distance 0.46- (Gene 1, Gene 2, Gene 4) joins Gene 3 at distance 1.62

This may result in ill-formed dendrograms. For an example, see the Java TreeView manual. A solution is to use the Euclidean or the city-block distance, or to use one of the other hierarchical clustering routines, which don't suffer from this issue regardless of the distance measure being used.

#### 4.1.2 Single Linkage Clustering

In Single Linkage Clustering the distance between two items
*x* and
*y* is the minimum of all pairwise distances between items contained in
*x* and
*y*. Unlike centroid linkage clustering, in single linkage clustering no further distances need to be calculated once the distance matrix is known.

In Cluster 3.0, as of version 1.29 the implementation of single linkage clustering is based on the SLINK algorithm (see Sibson, 1973). Whereas this algorithm yields the exact same clustering result as conventional single-linkage hierarchical clustering, it is much faster and more memory-efficient (being linear in the memory requirements, compared to quadratic for the conventional algorithm). Hence, single-linkage hierarchical clustering can be used to cluster large gene expression data sets, for which centroid-, complete-, and average-linkage fail due to lack of memory.

#### 4.1.3 Complete Linkage Clustering

In Complete Linkage Clustering the distance between two items
*x* and
*y* is the maximum of all pairwise distances between items contained in
*x* and
*y*. As in single linkage clustering, no other distances need to be calculated once the distance matrix is known.

#### 4.1.4 Average Linkage Clustering

In average linkage clustering, the distance between two items
*x* and
*y* is the mean of all pairwise distances between items contained in
*x* and
*y*.

#### 4.1.5 Weighting

Weighting: By default, all of the observations for a given item are treated equally. In
some cases you may want to give some observations more weight than others. For
example, if you have duplicate copies of a gene on your array, you might want to
downweight each individual copy when computing distances between arrays. You can
specify weights using the GWEIGHT (gene weight) and EWEIGHT (experiment weight)
parameters in the input file. By default all weights are set to 1.0. Thus, the actual formula,
with weights included, for the Pearson correlation of
*x* = {*x*1, *x*2, ..., *xn*}and
*y* = {*y*1, *y*2, ..., *yn*} with observation weights of
*w* = {*w*1, *w*2, ..., *wn*} is

|  |  |  |  |  |  |  |  |  |  |  |  |  |  |  |  |  |  |  |  |  |  |  |  |
| --- | --- | --- | --- | --- | --- | --- | --- | --- | --- | --- | --- | --- | --- | --- | --- | --- | --- | --- | --- | --- | --- | --- | --- |
| *r* = | |  |  |  |  |  |  |  |  |  |  |  |  |  |  |  |  |  | | --- | --- | --- | --- | --- | --- | --- | --- | --- | --- | --- | --- | --- | --- | --- | --- | --- | | |  |  |  |  |  |  |  |  |  |  |  |  |  |  |  |  | | --- | --- | --- | --- | --- | --- | --- | --- | --- | --- | --- | --- | --- | --- | --- | --- | | *n*  ∑  *i* = 1 | *w**i* | ( | |  | | --- | | *x**i* - *x* | | |  | | --- | |  | | | σ*x* | | ) | ( | |  | | --- | | *y**i* - *y* | | |  | | --- | |  | | | σ*y* | | ) | | | |  | | --- | |  | | | |  |  | | --- | --- | | *n*  ∑  *i* = 1 | *w**i* | | |

Note that when you are clustering rows (genes), you are using column (array) weights.
It is possible to compute weights as well based on a not entirely well understood function.
If you want to compute weights for clustering genes, select the check box in the Genes panel of the Hierarchical Clustering tab.   

This will expose a Weight Options dialog box in the Arrays panel (I realize this
placement is a bit counterintuitive, but it makes sense as you will see below).
The idea behind the Calculate Weights option is to weight each row (the same idea
applies to columns as well) based on the local density of row vectors in its vicinity, with a
high density vicinity resulting in a low weight and a low density vicinity resulting in a
higher weight. This is implemented by assigning a local density score
*L*(*i*) to each row
*i*.   

|  |  |  |  |  |  |  |  |  |  |  |
| --- | --- | --- | --- | --- | --- | --- | --- | --- | --- | --- |
| *L*(*i*) = |  | ∑  *j* with *d*(*i*,*j*) ≤ *k* | ( | |  | | --- | | *k* - *d*(*i*,*j*) | | |  | | --- | |  | | | *k* | | ) | *n* |

where the cutoff
*k* and the exponent
*n* are user supplied parameters. The weight for each row is
1/*L*. Note that
*L*(*i*) is always at least 1, since
*d*(*i*,*i*) = 0. Each other row that is within the distance
*k* of row
*i* increases
*L*(*i*) and decreases the weight. The larger
*d*(*i*,*j*), the less
*L*(*i*) is increased. Values of
*n* greater than 1 mean that the contribution to
*L*(*i*) drops off rapidly as
*d*(*i*,*j*) increases.

#### 4.1.6 Ordering of Output File

The result of a clustering run is a tree or pair of trees (one for genes one for arrays).
However, to visualize the results in TreeView, it is necessary to use this tree to reorder the
rows and/or columns in the initial datatable. Note that if you simply draw all of the node
in the tree in the following manner, a natural ordering of items emerges:
  


Thus, any tree can be used to generate an ordering. However, the ordering for any given
tree is not unique. There is a family of
2*N*-1ordering consistent with any tree of
*N*items;
you can flip any node on the tree (exchange the bottom and top branches) and you will
get a new ordering that is equally consistent with the tree. By default, when Cluster joins
two items, it randomly places one item on the top branch and the other on the bottom
branch. It is possible to guide this process to generate the best ordering consistent with
a given tree. This is done by using the GORDER (gene order) and EORDER (experiment
order) parameters in the input file, or by running a self-organizing map (see section
below) prior to clustering. By default, Cluster sets the order parameter for each
row/column to 1. When a new node is created, Cluster compares the order parameters of
the two joined items, and places the item with the smaller order value on the top branch.
The order parameter for a node is the average of the order parameters of its members.
Thus, if you want the gene order produced by Cluster to be as close as possible (without
violating the structure of the tree) to the order in your input file, you use the GORDER
column, and assign a value that increases for each row. Note that order parameters do not
have to be unique.

#### 4.1.7 Output Files

Cluster writes up to three output files for each hierarchical clustering run. The root
filename of each file is whatever text you enter into the Job Name dialog box. When you
load a file, Job Name is set to the root filename of the input file. The three output files are
JobName.cdt, JobName.gtr, JobName.atr
The .cdt (for clustered data table) file contains the original data with the rows and
columns reordered based on the clustering result. It is the same format as the input files,
except that an additional column and/or row is added if clustering is performed on genes
and/or arrays. This additional column/row contains a unique identifier for each
row/column that is linked to the description of the tree structure in the .gtr and .atr files.
The .gtr (gene tree) and .atr (array tree) files are tab-delimited text files that report on the
history of node joining in the gene or array clustering (note that these files are produced
only when clustering is performed on the corresponding axis). When clustering begins
each item to be clustered is assigned a unique identifier (e.g. GENE1X or ARRY42X — the
X is a relic from the days when this was written in Perl and substring searches were
used). These identifiers are added to the .cdt file. As each node is generated, it receives a
unique identifier as well, starting is NODE1X, NODE2X, etc. Each joining event is
stored in the .gtr or .atr file as a row with the node identifier, the identifiers of the two
joined elements, and the similarity score for the two joined elements. These files look like:  


|  |  |  |  |
| --- | --- | --- | --- |
| `NODE1X` | `GENE1X` | `GENE4X` | `0.98` |
| `NODE2X` | `GENE5X` | `GENE2X` | `0.80` |
| `NODE3X` | `NODE1X` | `GENE3X` | `0.72` |
| `NODE4X` | `NODE2X` | `NODE3X` | `0.60` |

  
The .gtr and/or .atr files are automatically read in TreeView when you open the
corresponding .cdt file.
