# Supplementary material for: A genome‐scale screen reveals context‐dependent ovarian cancer sensitivity to miRNA overexpression
Source: Mol Syst Biol. 2015 Dec 11;11(12):842. doi: 10.15252/msb.20156308 (PMC4704493; doi:10.15252/msb.20156308)
Supplement: Supplementary file 12 — Dataset EV8 [file MSB-11-842-s016.zip › Dataset_EV8/Cluster.app/Contents/Resources/html/index.html]

Cluster 3.0 for Windows, Mac OS X, Linux, Unix


# Cluster 3.0 for Windows, Mac OS X, Linux, Unix

Next: Contents


---

This is the manual for Cluster 3.0. Cluster was originally written by Michael Eisen while at Stanford University. We have modified the
*k*-means clustering algorithm in Cluster, and extended the algorithm for Self-Organizing Maps to include two-dimensional rectangular grids. The Euclidean distance and the city-block distance were added as new distance measures between gene expression data. The proprietary Numerical Recipes routines, which were used in the original version of Cluster/TreeView, have been replaced by open source software.

Cluster 3.0 is available for Windows, Mac OS X, Linux, and Unix.

November 5, 2002.  
Michiel de Hoon  
Human Genome Center, University of Tokyo.

- Introduction: The purpose of Cluster/TreeView.- Data: Loading, filtering and adjusting data in Cluster.- Distance: The distance/similarity measures that are available in Cluster.- Cluster: The various clustering algorithms implemented in Cluster.- Command: A command-line (non-GUI) version of Cluster 3.0 is now available.- TreeView: Visualize hierarchical clustering results with Java TreeView.- Development: Information on how to compile Cluster from the source code.- Bibliography: The bibliography provides references to background information on clustering techniques, as well as some examples of recent biological research in which clustering techniques are applied.- Contents
