# Supplementary material for: A genome‐scale screen reveals context‐dependent ovarian cancer sensitivity to miRNA overexpression
Source: Mol Syst Biol. 2015 Dec 11;11(12):842. doi: 10.15252/msb.20156308 (PMC4704493; doi:10.15252/msb.20156308)
Supplement: Supplementary file 12 — Dataset EV8 [file MSB-11-842-s016.zip › Dataset_EV8/Cluster.app/Contents/Resources/html/Introduction.html]

Introduction - Cluster 3.0 for Windows, Mac OS X, Linux, Unix


Next: Data,
Previous: Contents,
Up: Top


---

## 1 Introduction

Cluster and TreeView are programs that provide a computational and graphical
environment for analyzing data from DNA microarray experiments, or other genomic
datasets. The program Cluster can organize and analyze the data in a number of
different ways. TreeView allows the organized data to
be visualized and browsed.

This manual is intended as a reference for using the software, and not as a
comprehensive introduction to the methods employed. Many of the methods are
drawn from standard statistical cluster analysis. There are excellent textbooks
available on cluster analysis which are listed in the bibliography at the end.
The bibliography also contains citations for recent publications in the
biological sciences, especially genomics, that employ
methods similar to those used here.
