# Supplementary material for: A genome‐scale screen reveals context‐dependent ovarian cancer sensitivity to miRNA overexpression
Source: Mol Syst Biol. 2015 Dec 11;11(12):842. doi: 10.15252/msb.20156308 (PMC4704493; doi:10.15252/msb.20156308)
Supplement: Supplementary file 12 — Dataset EV8 [file MSB-11-842-s016.zip › Dataset_EV8/Cluster.app/Contents/Resources/html/KMeans.html]

KMeans - Cluster 3.0 for Windows, Mac OS X, Linux, Unix


Next: SOM,
Previous: Hierarchical,
Up: Cluster


---

### 4.2 The *k*-means Clustering Algorithm

The
*k*-means clustering algorithm is a simple, but popular, form of cluster analysis. The basic idea is that you start with a collection of items (e.g. genes) and some chosen number of clusters
(*k*) you want to find. The items are initially randomly assigned to a cluster. The
*k*-means clustering proceeds by repeated application of a two-step process where

1. the mean vector for all items in each cluster is computed;- items are reassigned to the cluster whose center is closest to the item.

Since the initial cluster assignment is random, different runs of the
*k*-means clustering algorithm may not give the same final clustering solution. To deal with this, the
*k*-means clustering algorithms is repeated many times, each time starting from a different initial clustering. The sum of distances within the clusters is used to compare different clustering solutions. The clustering solution with the smallest sum of within-cluster distances is saved.

The number of runs that should be done depends on how difficult it is to find the optimal solution, which in turn depends on the number of genes involved. Cluster therefore shows in the status bar how many times the optimal solution has been found. If this number is one, there may be a clustering solution with an even lower sum of within-cluster distances. The
*k*-means clustering algorithm should then be repeated with more trials. If the optimal solution is found many times, the solution that has been found is likely to have the lowest possible within-cluster sum of distances. We can then assume that the
*k*-means clustering procedure has then found the overall optimal clustering solution.

It should be noted that generally, the
*k*-means clustering algorithm finds a clustering solution with a smaller within-cluster sum of distances than the hierarchical clustering techniques.

The parameters that control
*k*-means clustering are

- the number of clusters
  (*k*);- the number of trials.

The output is simply an assignment of items to a cluster. The implementation here simply rearranges the rows and/or columns based on which cluster they were assigned to.
The output data file is JobName\_K\_GKg\_AKa.cdt, where \_GKg is included if genes were organized, and \_AKa is included if arrays were organized. Here, Kg and Ka represent the number of clusters for gene clustering and array clustering, respectively. This file contains the gene expression data, organized by cluster by rearranging the rows and columns. In addition, the files JobName\_K\_GKg.kgg and JobName\_K\_AKa.kag are created, containing a list of genes/arrays and the cluster they were assigned to.

Whereas
*k*-means clustering as implemented in Cluster 3.0 allows any of the eight distance measures to be used, we recommend using the Euclidean distance or city-block distance instead of the distance measures based on the Pearson correlation, for the same reason as in case of pairwise centroid-linkage hierarchical clustering. The distance measures based on the Pearson correlation effectively normalize the data vectors when calculating the distance, whereas no normalization is used when calculating the cluster centroid. To use
*k*-means clustering with a distance measure based on the Pearson correlation, it is better to first normalize the data appropriately (using the "Adjust Data" tab) before running the
*k*-means algorithm.

Cluster also implements a slight variation on
*k*-means clustering, known as
*k*-medians clustering, in which the median instead of the mean of items in a node are used. In a theoretical sense, it is best to use
*k*-means with the Euclidean distance, and
*k*-medoids with the city-block distance.
