# Supplementary material for: A genome‐scale screen reveals context‐dependent ovarian cancer sensitivity to miRNA overexpression
Source: Mol Syst Biol. 2015 Dec 11;11(12):842. doi: 10.15252/msb.20156308 (PMC4704493; doi:10.15252/msb.20156308)
Supplement: Supplementary file 12 — Dataset EV8 [file MSB-11-842-s016.zip › Dataset_EV8/Cluster.app/Contents/Resources/html/PCA.html]

PCA - Cluster 3.0 for Windows, Mac OS X, Linux, Unix


Previous: SOM,
Up: Cluster


---

### 4.4 Principal Component Analysis

Principal Component Analysis (PCA) is a widely used technique for analyzing multivariate data. A practical example of applying Principal Component Analysis to gene expression data is presented by Yeung and Ruzzo (2001).

In essence, PCA is a coordinate transformation in which each row in the data matrix is written as a linear sum over basis vectors called principal components, which are ordered and chosen such that each maximally explains the remaining variance in the data vectors. For example, an n \times 3 data matrix can be represented as an ellipsoidal cloud of n points in three dimensional space. The first principal component is the longest axis of the ellipsoid, the second principal component the second longest axis of the ellipsoid, and the third principal component is the shortest axis. Each row in the data matrix can be reconstructed as a suitable linear combination of the principal components. However, in order to reduce the dimensionality of the data, usually only the most important principal components are retained. The remaining variance present in the data is then regarded as unexplained variance.

The principal components can be found by calculating the eigenvectors of the covariance matrix of the data. The corresponding eigenvalues determine how much of the variance present in the data is explained by each principal component.

Before applying PCA, typically the mean is subtracted from each column in the data matrix. In the example above, this effectively centers the ellipsoidal cloud around its centroid in 3D space, with the principal components describing the variation of poins in the ellipsoidal cloud with respect to their centroid.

In Cluster, you can apply PCA to the rows (genes) of the data matrix, or to the columns (microarrays) of the data matrix. In each case, the output consists of two files. When applying PCA to genes, the names of the output files are JobName\_pca\_gene.pc.txt and JobName\_pca\_gene.coords.txt, where the former contains contains the principal components, and the latter contains the coordinates of each row in the data matrix with respect to the principal components. When applying PCA to the columns in the data matrix, the respective file names are JobName\_pca\_array.pc.txt and JobName\_pca\_array.coords.txt. The original data matrix can be recovered from the principal components and the coordinates.

As an example, consider this input file:

|  |  |  |  |
| --- | --- | --- | --- |
| `UNIQID` | `EXP1` | `EXP2` | `EXP3` |
| `GENE1` | `3` | `4` | `-2` |
| `GENE2` | `4` | `1` | `-3` |
| `GENE3` | `1` | `-8` | `7` |
| `GENE4` | `-6` | `6` | `4` |
| `GENE5` | `0` | `-3` | `8` |

Applying PCA to the rows (genes) of the data in this input file generates a coordinate file containing

|  |  |  |  |  |  |
| --- | --- | --- | --- | --- | --- |
| `UNIQID` | `NAME` | `GWEIGHT` | `13.513398` | `10.162987` | `2.025283` |
| `GENE1` | `GENE1` | `1.000000` | `6.280326` | `-2.404095` | `-0.760157` |
| `GENE2` | `GENE2` | `1.000000` | `4.720801` | `-4.995230` | `0.601424` |
| `GENE3` | `GENE3` | `1.000000` | `-8.755665` | `-2.117608` | `0.924161` |
| `GENE4` | `GENE4` | `1.000000` | `3.443490` | `8.133673` | `0.621082` |
| `GENE5` | `GENE5` | `1.000000` | `-5.688953` | `1.383261` | `-1.386509` |

where the first line shows the eigenvalues of the principal components, and a prinpical component file containing

|  |  |  |  |
| --- | --- | --- | --- |
| `EIGVALUE` | `EXP1` | `EXP2` | `EXP3` |
| `MEAN` | `0.400000` | `0.000000` | `2.800000` |
| `13.513398` | `0.045493` | `0.753594` | `-0.655764` |
| `10.162987` | `-0.756275` | `0.454867` | `0.470260` |
| `2.025283` | `-0.652670` | `-0.474545` | `-0.590617` |

with the eigenvalues of the principal components shown in the first column. From this principal component decomposition, we can regenerate the original data matrix as follows:

|  |  |  |  |  |
| --- | --- | --- | --- | --- |
| ⎛ | 6.280326 | -2.404095 | -0.760157 | ⎞ |
| ⎜ | 4.720801 | -4.995230 | 0.601424 | ⎟ |
| ⎜ | -8.755665 | -2.117608 | 0.924161 | ⎟ |
| ⎜ | 3.443490 | 8.133673 | 0.621082 | ⎟ |
| ⎝ | -5.688953 | 1.383261 | -1.386509 | ⎠ |

|  |
| --- |
|  |
|  |
| · |

|
|  |
| ⎛ | 0.045493 | 0.753594 | -0.655764 | ⎞ |
| ⎜ | -0.756275 | 0.454867 | 0.470260 | ⎟ |
| ⎝ | -0.652670 | -0.474545 | -0.590617 | ⎠ |

|  |
| --- |
|  |
|  |
| + |

|  |  |  |  |  |
| --- | --- | --- | --- | --- |
| ⎛ | 0.4 | 0.0 | 2.8 | ⎞ |
| ⎜ | 0.4 | 0.0 | 2.8 | ⎟ |
| ⎜ | 0.4 | 0.0 | 2.8 | ⎟ |
| ⎜ | 0.4 | 0.0 | 2.8 | ⎟ |
| ⎝ | 0.4 | 0.0 | 2.8 | ⎠ |

|  |
| --- |
|  |
|  |
| = |

|  |  |  |  |  |
| --- | --- | --- | --- | --- |
| ⎛ | 3 | 4 | -2 | ⎞ |
| ⎜ | 4 | 1 | -3 | ⎟ |
| ⎜ | 1 | -8 | 7 | ⎟ |
| ⎜ | -6 | 6 | 4 | ⎟ |
| ⎝ | 0 | -3 | 8 | ⎠ |

Note that the coordinate file JobName\_pca\_gene.coords.txt is a valid input file to Cluster 3.0. Hence, it can be loaded into Cluster 3.0 for further analysis, possibly after removing columns with low eigenvalues.
