# Supplementary material for: A genome‐scale screen reveals context‐dependent ovarian cancer sensitivity to miRNA overexpression
Source: Mol Syst Biol. 2015 Dec 11;11(12):842. doi: 10.15252/msb.20156308 (PMC4704493; doi:10.15252/msb.20156308)
Supplement: Supplementary file 12 — Dataset EV8 [file MSB-11-842-s016.zip › Dataset_EV8/Cluster.app/Contents/Resources/html/SOM.html]

SOM - Cluster 3.0 for Windows, Mac OS X, Linux, Unix


Next: PCA,
Previous: KMeans,
Up: Cluster


---

### 4.3 Self-Organizing Maps

Self-Organizing Maps (SOMs) is a method of cluster analysis that are somewhat related to
*k*-meansclustering. SOMs were invented in by Teuvo Kohonen in the early 1980s, and
have recently been used in genomic analysis (see Chu 1998, Tamayo 1999 and Golub
1999 in references). The Tamayo paper contains a simple explanation of the methods. A
more detailed description is available in the book by Kohonen, Self-Organizing Maps,
1997.

The current implementation varies slightly from that of Tamayo et al., in that it restricts
the analysis one-dimensional SOMs along each axis, as opposed to a two-dimensional
network. The one-dimensional SOM is used to reorder the elements on whichever axes
are selected. The result is similar to the result of k-means clustering, except that, unlike in k-means clustering,
the nodes in a SOM are ordered. This tends to result in a relatively smooth
transition between groups.

The options for SOMs are

- whether or not you will organize each axis;- the number of nodes for each axis (the default is
    *n*1/4,where
    *n*is the number of items; the total number of clusters is then equal to the square root of the number of items);- the number of iterations to be run.

The output file is of the form JobName\_SOM\_GXg-Yg\_AXa-Ya.txt, where GXg-Yg is
included if genes were organized, and AXg-Yg is included if arrays were organized. X and
Y represent the dimensions of the corresponding SOM.
Up to two additional files (.gnf and .anf) are written containing the vectors for
the SOM nodes.

In previous versions of Cluster, only one-dimensional SOMs were supported.
The current version of the Cluster introduces two-dimensional SOMs.

SOMs and hierarchical clustering:
Our original use of SOMs (see Chu et al., 1998) was
motivated by the desire to take advantage of the properties of both SOMs and hierarchical
clustering. This was accomplished by first computing a one dimensional SOM, and using
the ordering from the SOM to guide the flipping of nodes in the hierarchical tree. In
Cluster, after a SOM is run on a dataset, the GORDER and/or EORDER fields are set to
the ordering from the SOM so that, for subsequent hierarchical clustering runs, the output
ordering will come as close as possible to the ordering in the SOM without violating the
structure of the tree.
