# Supplementary material for: A genome‐scale screen reveals context‐dependent ovarian cancer sensitivity to miRNA overexpression
Source: Mol Syst Biol. 2015 Dec 11;11(12):842. doi: 10.15252/msb.20156308 (PMC4704493; doi:10.15252/msb.20156308)
Supplement: Supplementary file 12 — Dataset EV8 [file MSB-11-842-s016.zip › Dataset_EV8/Cluster.app/Contents/Resources/html/TreeView.html]

TreeView - Cluster 3.0 for Windows, Mac OS X, Linux, Unix


Next: Development,
Previous: Command,
Up: Top


---

## 6 TreeView

TreeView is a program that allows interactive graphical analysis of the results from Cluster. TreeView reads in matching \*.cdt and \*.gtr, \*.atr, \*.kgg, or \*.kag files produced by Cluster.
We recommend using the Java program Java TreeView, which is based on the original TreeView. Java TreeView was written by Alok Saldanha at Stanford University; it can be downloaded from http://jtreeview.sourceforge.net/. Java TreeView runs on Windows, Macintosh, Linux, and Unix computers, and can show both hierarchical and
*k*-meansresults.
