# Supplementary material for: A genome‐scale screen reveals context‐dependent ovarian cancer sensitivity to miRNA overexpression
Source: Mol Syst Biol. 2015 Dec 11;11(12):842. doi: 10.15252/msb.20156308 (PMC4704493; doi:10.15252/msb.20156308)
Supplement: Supplementary file 13 — Dataset EV9 [file MSB-11-842-s017.zip › Dataset_EV9/Dataset EV9 README.rtf]

Dataset EV9:A unidirectional cluster using Euclidian distance of miRNA expression values for PEO1 and PEO4 normalized to HOSE cell expression. See Dataset EV6 for values. 
