# Supplementary material for: A genome‐scale screen reveals context‐dependent ovarian cancer sensitivity to miRNA overexpression
Source: Mol Syst Biol. 2015 Dec 11;11(12):842. doi: 10.15252/msb.20156308 (PMC4704493; doi:10.15252/msb.20156308)
Supplement: Supplementary file 18 — Source Data for Figure 3 [file MSB-11-842-s006.zip › Source Data Fig 3/Source Data Figure 3G.pdf]

Uncut Blot for Figure 3G

T = treated with miR-517a
C = treated with miNC

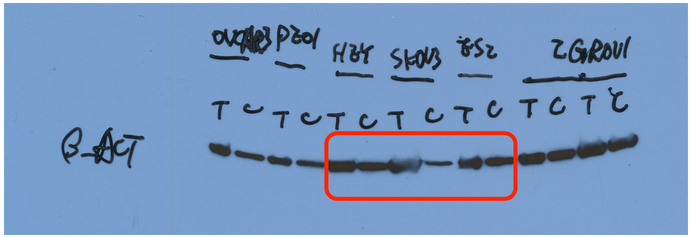

beta-actin

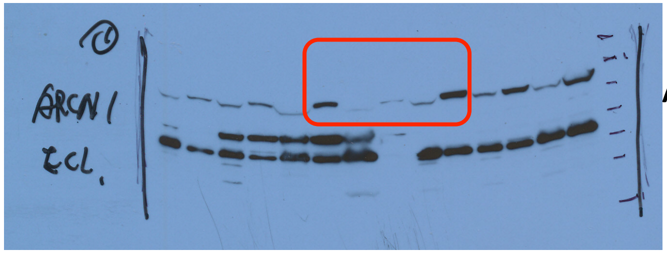

Archain 1

IGROV1 A2780 SKOV3 PEO4 HEY OVCAR3 PEO1 ES2

CT CT CT CT CT CT TC TC

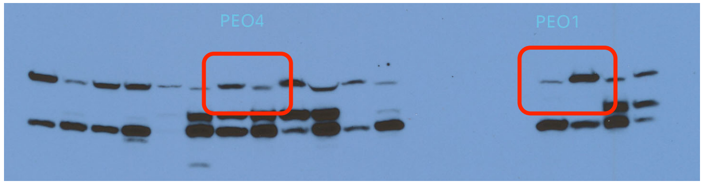

Archain 1

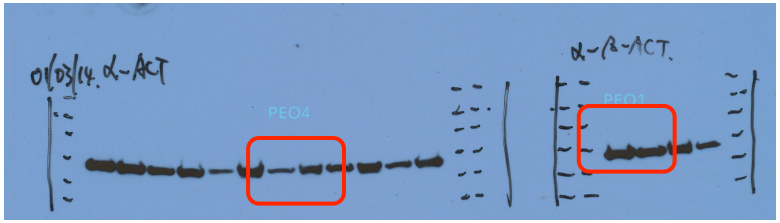

beta-actin

C T C T C T C T

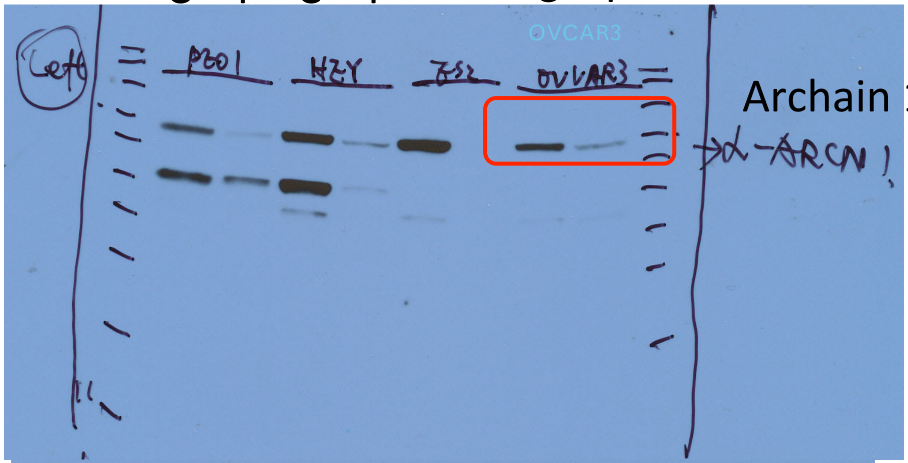

Archain 1

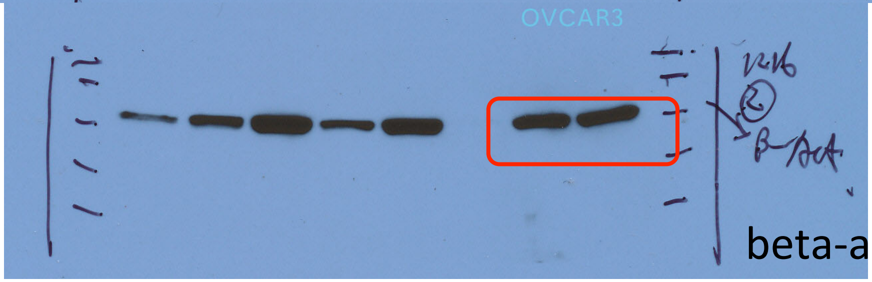

beta-actin

Lanes used highlighted in red
