# Supplementary material for: A genome‐scale screen reveals context‐dependent ovarian cancer sensitivity to miRNA overexpression
Source: Mol Syst Biol. 2015 Dec 11;11(12):842. doi: 10.15252/msb.20156308 (PMC4704493; doi:10.15252/msb.20156308)

Supplementary Dataset 10

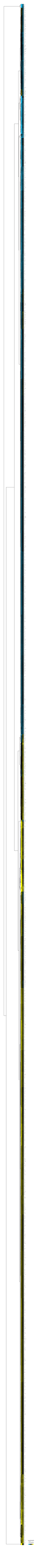

Supplement: Supplementary file 14 — Dataset EV10 [file Dataset EV10.pdf › Dataset_EV10/Dataset EV10.pdf]
